# Difunctionalization of alkenes with iodine and *tert*-butyl hydroperoxide (TBHP) at room temperature for the synthesis of 1-(*tert*-butylperoxy)-2-iodoethanes

**DOI:** 10.3762/bjoc.13.200

**Published:** 2017-09-28

**Authors:** Hao Wang, Cui Chen, Weibing Liu, Zhibo Zhu

**Affiliations:** 1 Integrated Hospital of Traditional Chinese Medicine, Southern Medical University, Guangzhou, 510315, P. R. China. Fax: +86- 20- 6164 8538; Tel: +86- 20 -62789464; 2 College of Chemical Engineering, Guangdong University of Petrochemical Technology, 2 Guandu Road, Maoming 525000, P. R. China. Fax: +86-668-2923575; Tel: +86-668-2923956

**Keywords:** difunctionalization of alkenesiodine, iodination–peroxidation reaction, TBHP

## Abstract

We developed a direct vicinal difunctionalization of alkenes with iodine and TBHP at room temperature. This iodination and peroxidation in a one-pot synthesis produces 1-(*tert*-butylperoxy)-2-iodoethanes, which are inaccessible through conventional synthetic methods. This method generates multiple radical intermediates in situ and has excellent regioselectivity, a broad substrate scope and mild conditions. The iodine and peroxide groups of 1-(*tert*-butylperoxy)-2-iodoethanes have several potential applications and allow further chemical modifications, enabling the preparation of synthetically valuable molecules.

## Introduction

**Scheme 1:**
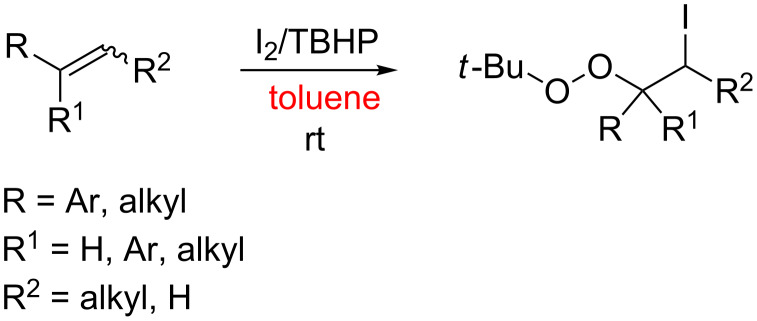
Synthesis of 1-(*tert*-butylperoxy)-2-iodoethanes.

Alkenes have attracted considerable interest in recent years as abundant, simple chemical feedstocks and organic molecules, owing to their potential for extensive application in organic syntheses. Approaches for the efficient, regio- and chemoselective difunctionalization of alkenes have been developed that are attractive for rapidly building complex difunctionalized molecules from simple starting materials in a single operation [1–6]. Traditional studies have mainly focused on transition-metal-catalyzed direct vicinal difunctionalization of alkenes by installing two substituents across the C=C double bond to form two new bonds or two new functional groups [7–9], such as dioxygenation [10–11], dihydroxylation [10,12], bisperoxidation [13], oxyamidation [14], aminohydroxylation [15], oxyphosphorylation [16], deamination [17] and carbonylation–peroxidation [18]. Several very recent reports have pertained to metal-free catalysts for difunctionalization of alkenes such as UV light [19–20], hypervalent iodine reagents [21–22], acids [23], organoammonium iodides [24] and iodine [25]. These catalysts are often employed in combination with a peroxide and generally produce an organoperoxide. Organic peroxides are important and useful compounds because of their unique chemical and biological properties [26–27]. Organoperoxides have wide applications in the field of organic synthesis, as radical initiators, oxidants that replace transition metal oxidants, and key reactive intermediates in diverse organic synthesis reactions [28–30], as well as in medicinal chemistry and pharmacology as medicines and therapeutic drugs [31–32]. Although many methods have been developed to synthesize peroxides [33–35], they have a tendency to decompose because the peroxy (–OO–) bond is easily cleaved and peroxides are highly sensitivity to reducing agents. New and general methods to construct peroxides are still highly desirable and valuable, and highly regioselective and efficient syntheses of peroxides with structural control are still difficult to achieve. Herein, we report a metal-free iodination–peroxidation reaction for the direct vicinal difunctionalization of alkenes with iodine and *tert*-butyl hydroperoxide (TBHP) at room temperature to synthesize 1-(*tert*-butylperoxy)-2-iodoethanes that are inaccessible via conventional synthetic routes (Scheme 1). To the best of our knowledge, β-iodoalkyl *tert*-butyl peroxides are important organic compounds due to their unique structural features, which make them available to serve as starting materials for a wide range of organic oxidations to access other oxygenated products [36].

## Results and Discussion

A pilot reaction setup comprised of styrene (**1a**, 208 mg, 2.0 mmol) in the presence of I_2_ (1.0 equiv), TBHP (2.0 equiv), and CH_3_CN (2.0 mL) was investigated to determine the optimal reaction conditions. Stirring this mixture at room temperature for 12 h afforded the desired product (2-iodo-1-(*tert*-butylperoxy)ethyl)benzene (**2a**) in 45% yield, which was further improved to 59% by extending the reaction time to 24 h (Table 1, entries 1–3). The reaction was quite sensitive to the solvent medium (Table 1, entries 2, 4–8). Among the various solvents examined, toluene proved to be the most suitable solvent, furnishing **2a** in 72% yield (Table 1, entry 8). Further screening studies were conducted by altering the amounts of I_2_ and TBHP to find the optimum reaction conditions. Notably, decreasing the amount of I_2_ to 0.5 equiv decreased the yield to 51% (Table 1, entry 9). An excess of 3.0 equiv of TBHP or more gave the highest product yields (Table 1, entries 8, 10 and 11).

**Table 1:** Optimization studies^a^.

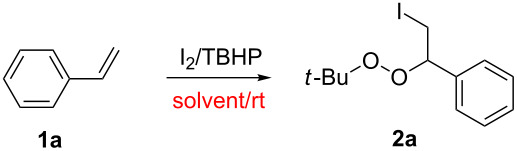			

Entry	Solvent	Time (h)	Yield (%)^b^

1	CH_3_CN	12	45
2	CH_3_CN	24	59
3	CH_3_CN	36	59
4	DCE	24	31
5	dioxane	24	57
6	DMF	24	trace
7	DMSO	24	trace
8	toluene	24	72
9^c^	toluene	24	51
10^d^	toluene	24	86
11^e^	toluene	24	86

^a^Unless otherwise specified, all reactions were carried out on **1a** 0.5 mmol scale, iodine 1.0 equiv, *tert*-butyl hydroperoxide (TBHP) 2.0 equiv, solvent 2.0 mL; ^b^yield calculated by GC; ^c^I_2_: 0.5 equiv; ^d^TBHP: 3.0 equiv; ^e^TBHP: 4.0 equiv.

**Scheme 2:**
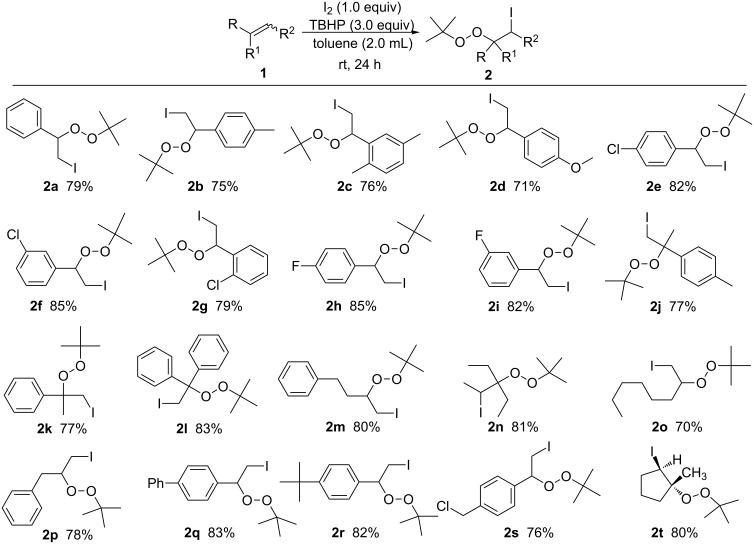
Direct vicinal difunctionalization of alkenes. All reactions were carried out on a 2.0 mmol scale using toluene (2.0 mL) as the solvent and all the listed yields are isolated yields.

A variety of substituted alkenes were then tested under the optimal reaction conditions identified above. Styrenes (R = Ar; R^1^ = R^2^ = H) **1a–i** bearing functional groups with different electronic properties on the phenyl ring were all tolerated and did not substantially alter the reaction efficiency (Scheme 2). Substrates bearing electron-withdrawing substituents (i.e., fluoro- and chloro-) performed slightly more effectively in this reaction than those bearing electron-donating substituents (i.e., methyl-, phenyl-, chloromethyl-, *tert*-butyl- and methoxy-), and afforded the corresponding products in relatively high yields.

Notably, the yield of this iodination–peroxidation reaction appeared to be unaffected by the position of the same substituent on the phenyl ring. For example, all pairs of 1-methyl-4-vinylbenzene and 1,4-dimethyl-2-vinylbenzene, 1-chloro-4-vinylbenzene and 1-chloro-3-vinylbenzene, 1-fluoro-4-vinylbenzene and 1-fluoro-3-vinylbenzene gave almost identical yields of the corresponding products. In addition, the inclusion of different R^1^ and R^2^ groups on styrenes, such as 1,1-disubstituted/1,2-disubstituted/1,1,2-trisubstituted alkenes (1-(prop-1-en-2-yl)benzene, 1-methyl-4-(prop-1-en-2-yl)benzene and 1,1-diphenylethene), had no discernible impact on the outcome of this iodination–peroxidation reaction. The scope of this reaction was further extended to a series of chain alkenes, such as 1-(but-3-enyl)benzene), 1-allylbenzene, oct-1-ene and 3-ethylpent-2-ene, which all reacted as anticipated to give the corresponding products in moderate to excellent isolated yields (70−81%). It is noteworthy that monosubstituted and 1,1-disubstituted terminal alkenes **1j-l**, as well 1,2,2-trisubstituted internal alkenes 3-ethylpent-2-ene (**1n**) and 1-methylcyclopent-1-ene (**1t**) under the developed conditions, all result the anticipated products with excellent yields. Especially, according to the detection of NMR and NOE analysis, the substrate 1-methylcyclopent-1-ene (**1t**) affords (1*R*,2*R*)-1-(*tert*-butylperoxy)-2-iodo-1-methylcyclopentane (**2t**) as the only product. And even more exciting this reaction was easily scaled up to 10 mmol with no obvious loss in product formation efficiency.

**Scheme 3:**
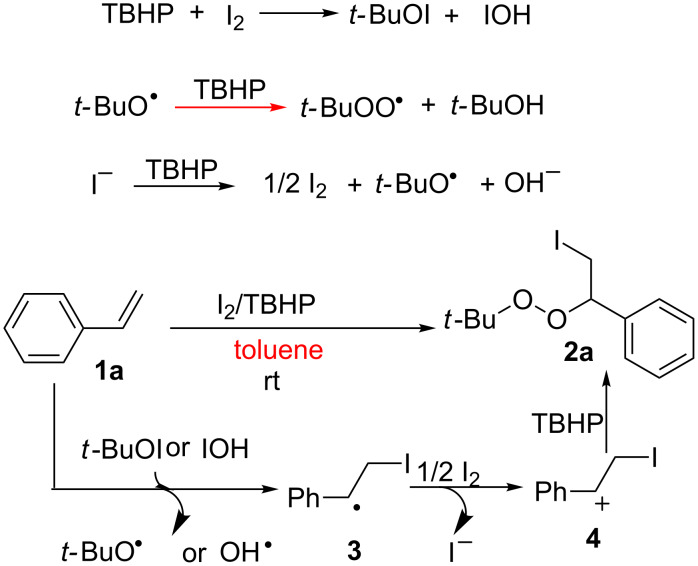
Possible reaction mechanism.

We investigated the addition of the radical inhibitor TEMPO (2,2,6,6-tetramethylpiperididine-*N*-oxyl) to gain an insight into the mechanism of this reaction. The reaction was completely inhibited in the presence of TEMPO, suggesting that a radical pathway may be operating in this reaction. Therefore, we propose a possible mechanism for the reaction in Scheme 3, exemplified by the formation of **2a**, based on the aforementioned results and previous literature [36–39]. The process commences with the formation of *t*-BuOI and HOI from the initial reaction of TBHP with I_2_ [40]. The subsequent iodination reaction could proceed via a homolytic attack involving *t*-BuOI and HOI to generate intermediate **3**, *tert*-butoxyl radicals and hydroxyl radicals[41]. In the presence of TBHP, a fast reaction between *tert*-butoxyl radicals and TBHP take place, leading to the formation of *tert*-butylperoxyl radicals and tertiary butanol [41–42]. Subsequently, the radical **3** can be further oxidized into cation **4** with I_2_ [24,43–44]. The iodide ion is then reoxidized with TBHP to regenerate I_2_ and *tert*-butoxyl radicals [24,45]. Finally, cation **4** can be attacked by TBHP to give the final product **2a**.

## Conclusion

In summary, we have established a metal-free process at room temperature for the direct vicinal difunctionalization of alkenes with iodine and TBHP to synthesize 1-(*tert*-butylperoxy)-2-iodoethanes. This procedure is a simple and high-yielding method with excellent regioselectivity for iodination and peroxidation of the C=C double bond of alkenes and shows good functional group compatibility. Furthermore, the mild reaction conditions of this methodology and the ease of further modification of the iodine and peroxide groups in 1-(*tert*-butylperoxy)-2-iodoethanes indicate that this procedure has good potential for application in the fields of organic synthesis, medicinal chemistry and pharmacology. Further work toward expanding this protocol and investigations into the difunctionalization of alkenes with other electrophiles is currently underway in our laboratory, and the results will be reported in due course.

## Supporting Information

File 1 Full experimental details and copies of NMR spectral data.
