# Supplementary material for: Difunctionalization of alkenes with iodine and tert-butyl hydroperoxide (TBHP) at room temperature for the synthesis of 1-(tert-butylperoxy)-2-iodoethanes
Source: Beilstein J Org Chem. 2017 Sep 28;13:2023–7. doi: 10.3762/bjoc.13.200 (PMC5629392; doi:10.3762/bjoc.13.200)
Supplement: File 1 — Full experimental details and copies of NMR spectral data. [file Beilstein_J_Org_Chem-13-2023-s001.pdf]

Supporting Information

for

Difunctionalization of alkenes with iodine and *tert*-butyl hydroperoxide (TBHP) at room temperature for the synthesis of 1-(*tert*-butylperoxy)-2-iodoethanes

Hao Wang[1], Cui Chen[2], Weibing Liu*[,2] and Zhibo Zhu*[,1]

Address: [1]Integrated Hospital of Traditional Chinese Medicine, Southern Medical University, Guangzhou, 510315, P. R. China. Fax: +86- 20- 6164 8538; Tel: +86- 20 -62789464 and [2]College of Chemical Engineering, Guangdong University of Petrochemical Technology, 2 Guandu Road, Maoming 525000, P. R. China. Fax: +86-668-2923575; Tel: +86-668-2923956

Email: Zhibo Zhu - zhuzb676@smu.edu.cn; Weibing Liu - lwb409@gdupt.edu.cn.

*Corresponding author

Full experimental details and copies of NMR spectral data

General Information. All the reactions were carried out at room temperature for 24 h in a round-bottom flask equipped with a magnetic stir bar. Unless otherwise stated, all reagents and solvents were purchased from commercial suppliers and used without further purification. ^{1}H NMR and ^{13}C NMR spectra were recorded on a 400 MHz spectrometer in solutions of $CDCl_3$

using tetramethylsilane as the internal standard; δ values are given in ppm, and coupling constants (J) in Hz. HR-MS were obtained on a Q-TOF micro spectrometer.

Typical procedure: 1-(1-(*tert*-butylperoxy)-2-iodoethyl)benzene (2a). A mixture of styrene (**1a**) (208 mg, 2.0 mmol), I_2 (508 mg, 2.0 mmol), TBHP (774 mg, 6.0 mmol, 70% in water), and toluene (2.0 mL) was added successively in a round-bottom flask, and the resulting solution was stirred for 24 h at room temperature. The mixture was purified by column chromatography on silica gel to afford product **2a** with PE/ethyl acetate = 20/1 as the eluent.

1-(1-(*tert*-Butylperoxy)-2-iodoethyl)benzene (2a) [1]

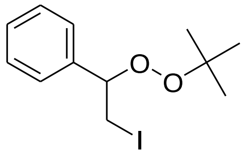

Yield: 79%; ^{1}H NMR ($CDCl_3$, 400 Hz) δ 7.40 (m, 5H), 4.88 (d, J = 6.0 Hz, 1H), 4.55 (dd, J = 7.2 Hz, J = 6.8 Hz, 1H), 3.64 (dd, J = 10.4 Hz, J = 6.4 Hz, 1H), 3.64 (dd, J = 9.6 Hz, J = 7.2 Hz, 1H), 1.28 (s, 9H); ^{13}C NMR ($CDCl_3$, 100 Hz) δ 138.2, 128.6, 128.4, 127.1, 85.3, 81.1, 26.5, 6.38.

1-(1-(*tert*-Butylperoxy)-2-iodoethyl)-4-methylbenzene (2b)

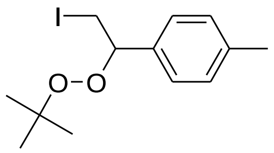

Yield: 75%; Pale yellow oil; ^{1}H NMR ($CDCl_3$, 400 Hz) δ 7.33 (d, J = 8.0 Hz, 2H), 7.27 (d, J = 8.0 Hz, 2H), 4.55 (dd, J = 7.2 Hz, J = 6.8 Hz, 1H), 3.74 (dd, J = 10.4 Hz, J = 6.0 Hz, 1H), 3.64 (dd, J = 10.4 Hz, J = 7.6 Hz, 1H), 2.44 (s, 3H), 1.37 (s, 9H); ^{13}C NMR ($CDCl_3$, 100 Hz) δ 138.5, 135.2, 129.2, 127.2, 85.3, 81.0, 26.6, 21.4, 6.76; HRMS (ESI): calcd for $C_{13}H_{19}INaO_2$: [M+Na$^+$] 357.0322, found 357.0329.

2-(1-(*tert*-Butylperoxy)-2-iodoethyl)-1,4-dimethylbenzene (2c)

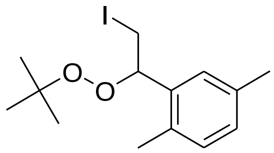

Yield: 76%; Colorless oil; ^{1}H NMR ($CDCl_3$, 400 Hz) δ 7.20 (s, 1H), 7.27 (m, 2H), 5.33 (dd, J = 7.0 Hz, J = 6.8 Hz, 1H), 3.67 (dd, J = 10.0 Hz, J = 6.4 Hz, 1H), 3.41 (dd, J = 10.4 Hz, J = 7.6 Hz, 1H), 2.40 (s, 3H), 2.37 (s, 3H), 1.32 (s, 9H); ^{13}C NMR ($CDCl_3$, 100 Hz) δ 136.2, 135.5, 132.9, 130.4, 129.2, 126.7, 81.8, 80.9, 26.6, 21.2, 19.1, 5.75; HRMS (ESI): calcd for $C_{14}H_{21}INaO_2$: [M+Na$^+$] 371.0478, found

371.0495.

1-(1-(*tert*-Butylperoxy)-2-iodoethyl)-4-methoxybenzene (2d)

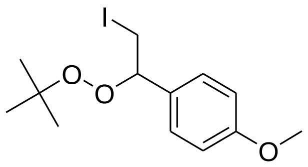

Yield: 71%; Pale yellow oil; ^{1}H NMR ($CDCl_3$, 400 Hz) δ 7.29 (d, *J* = 8.0 Hz, 2H), 6.92 (d, *J* = 8.0 Hz, 2H), 4.99 (dd, *J* = 7.0 Hz, *J* = 6.8 Hz, 1H), 3.83 (s, 3H), 3.70 (dd, *J* = 10.0 Hz, *J* = 6.4 Hz, 1H), 3.39 (dd, *J* = 10.4 Hz, *J* = 7.6 Hz, 1H), 1.28 (s, 9H); ^{13}C NMR ($CDCl_3$, 100 Hz) δ 159.8, 130.0, 128.5, 113.8, 85.1, 81.0, 55.2, 26.4, 6.71; HRMS (ESI): calcd for $C_{13}H_{19}InaO_3$: [M+Na$^+$] 373.0271, found 373.0279.

1-(1-(*tert*-Butylperoxy)-2-iodoethyl)-4-chlorobenzene (2e)

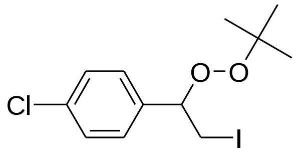

Yield: 82%; Pale yellow oil; ^{1}H NMR ($CDCl_3$, 400 Hz) δ 7.38 (d, *J* = 8.0 Hz, 2H), 7.30 (d, *J* = 8.0 Hz, 2H), 5.00 (dd, *J* = 7.0 Hz, *J* = 6.8 Hz, 1H), 3.59 (dd, *J* = 10.0 Hz, *J* = 6.4 Hz, 1H), 3.36 (dd, *J* = 10.4 Hz, *J* = 7.6 Hz, 1H), 1.28 (s, 9H); ^{13}C NMR ($CDCl_3$, 100 Hz) δ 136.8, 134.4, 128.7, 128.4, 84.5, 81.2, 26.5, 5.91; HRMS (ESI): calcd for $C_{12}H_{16}ClINaO_2$: [M+Na$^+$] 376.9776, found 376.9788.

1-(1-(*tert*-Butylperoxy)-2-iodoethyl)-3-chlorobenzene (2f)

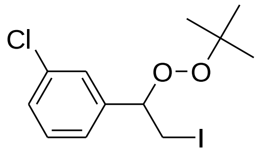

Yield: 85%; Pale yellow oil; ^{1}H NMR ($CDCl_3$, 400 Hz) δ 7.36 (s, 1H), 7.31 (m, 2H), 7.25 (m, 1H), 4.98 (dd, *J* = 7.0 Hz, *J* = 6.8 Hz, 1H), 3.57 (dd, *J* = 10.0 Hz, *J* = 6.4 Hz, 1H), 3.36 (dd, *J* = 10.4 Hz, *J* = 7.6 Hz, 1H), 1.29 (s, 9H); ^{13}C NMR ($CDCl_3$, 100 Hz) δ 140.5, 134.3, 129.7, 128.7, 127.1, 125.2, 84.5, 81.3, 26.5, 5.59; HRMS (ESI): calcd for $C_{12}H_{16}ClINaO_2$: [M+Na$^+$] 376.9776, found 376.9784.

1-(1-(*tert*-Butylperoxy)-2-iodoethyl)-2-chlorobenzene (2g)

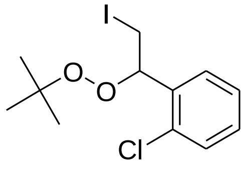

Yield: 79%; Pale yellow oil; ^{1}H NMR ($CDCl_3$, 400 Hz) δ 7.52 (dd, *J* = 2.0 Hz, *J* = 7.6 Hz, 1H), 7.35 (m,

3H), 4.45 (dd, J = 7.0 Hz, J = 6.8 Hz, 1H), 3.54 (dd, J = 10.0 Hz, J = 6.4 Hz, 1H), 3.39 (dd, J = 10.4 Hz, J = 7.6 Hz, 1H), 1.33 (s, 9H); ^{13}C NMR ($CDCl_3$, 100 Hz) δ 136.7, 132.7, 129.5, 129.4, 127.8, 126.9, 81.4, 80.9, 26.5, 4.41; HRMS (ESI): calcd for $C_{12}H_{16}ClINaO_2$: [M+Na$^+$] 376.9776, found 376.9778.

1-(1-(*tert*-Butylperoxy)-2-iodoethyl)-4-fluorobenzene (2h)

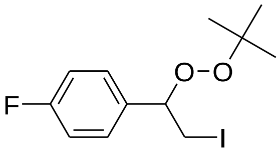

Yield: 85%; Colorless oil; ^{1}H NMR ($CDCl_3$, 400 Hz) δ 7.32 (d, Hz, J = 7.6 Hz, 2H), 7.07 (d, Hz, J = 7.6 Hz, 2H), 5.00 (dd, J = 7.0 Hz, J = 6.8 Hz, 1H), 3.62 (dd, J = 10.0 Hz, J = 6.4 Hz, 1H), 3.36 (dd, J = 10.4 Hz, J = 7.6 Hz, 1H), 1.27 (s, 9H); ^{13}C NMR ($CDCl_3$, 100 Hz) δ 161.8 (d, $^1J_{C\text{-}F}$ = 182.4 Hz), 134.0 (d, $^4J_{C\text{-}F}$ = 12.8 Hz), 128.8 (d, $^3J_{C\text{-}F}$ = 32.8 Hz), 115.4 (d, $^2J_{C\text{-}F}$ = 85.6 Hz), 84.6, 81.1, 26.5, 6.2; HRMS (ESI): calcd for $C_{12}H_{16}FINaO_2$: [M+Na$^+$] 361.0071, found 361.0079.

1-(1-(*tert*-Butylperoxy)-2-iodoethyl)-3-fluorobenzene (2i)

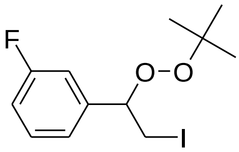

Yield: 82%; Colorless oil; ^{1}H NMR ($CDCl_3$, 400 Hz) δ 7.33 (m, 1H), 7.08 (m, 3H), 5.00 (dd, J = 7.0 Hz, J = 6.8 Hz, 1H), 3.56 (dd, J = 10.0 Hz, J = 6.4 Hz, 1H), 3.36 (dd, J = 10.4 Hz, J = 7.6 Hz, 1H), 1.28 (s, 9H); ^{13}C NMR ($CDCl_3$, 100 Hz) δ 163.9 (d, $^1J_{C\text{-}F}$ = 179.2 Hz), 141.0 (d, $^3J_{C\text{-}F}$ = 28.0 Hz), 129.9 (d, $^3J_{C\text{-}F}$ = 32.4 Hz), 122.7 (d, $^4J_{C\text{-}F}$ = 11.6 Hz), 115.5 (d, $^2J_{C\text{-}F}$ = 84.0 Hz), 113.9 (d, $^2J_{C\text{-}F}$ = 84.0 Hz), 84.4, 81.2, 26.5, 5.6; HRMS (ESI): calcd for $C_{12}H_{16}FINaO_2$: [M+Na$^+$] 361.0071, found 361.0088.

1-(2-(*tert*-Butylperoxy)-1-iodopropan-2-yl)-4-methylbenzene (2j)

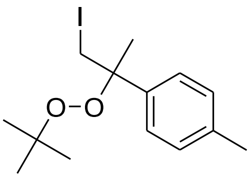

Yield: 77%; Colorless oil; ^{1}H NMR ($CDCl_3$, 400 Hz) δ 7.41 (d, J = 8.0 Hz, 2H), 7.24 (d, J = 8.0 Hz, 2H), 3.70 (s, 2H), 2.43 (s, 3H), 1.86 (s, 3H), 1.38 (s, 9H); ^{13}C NMR ($CDCl_3$, 100 Hz) δ 139.3, 137.2, 128.9, 125.9, 81.1, 79.7, 26.8, 24.4, 21.2, 15.8; HRMS (ESI): calcd for $C_{14}H_{21}INaO_2$: [M+Na$^+$] 371.0478, found 371.0465.

1-(2-(*tert*-Butylperoxy)-1-iodopropan-2-yl)benzene (2k)

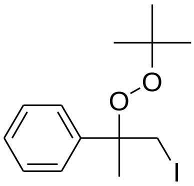

Yield: 77%; Colorless oil; ^{1}H NMR ($CDCl_3$, 400 Hz) δ 7.54 (d, J = 8.0 Hz, 2H), 7.45 (t, J = 8.0 Hz, 2H), 7.38 (t, J = 8.0 Hz, 1H), 3.73 (s, 2H), 1.89 (s, 3H), 1.41 (s, 9H); ^{13}C NMR ($CDCl_3$, 100 Hz) δ 142.3, 128.2, 127.6, 125.9, 81.2, 79.7, 26.9, 24.5, 15.6; HRMS (ESI): calcd for $C_{13}H_{19}INaO_2$: [M+Na$^+$] 357.0322, found 357.0337.

1-(*tert*-Butylperoxy)-2-iodo-1,1-diphenylethane (2l) [1]

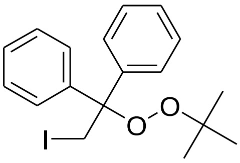

Yield: 83%; ^{1}H NMR ($CDCl_3$, 400 Hz) δ 7.44 (m, 4H), 7.35 (m, 6H), 4.39 (s, 2H), 1.35 (s, 9H); ^{13}C NMR ($CDCl_3$, 100 Hz) δ 142.6, 127.8, 127.4, 127.1, 84.4, 79.9, 26.9, 15.3.

1-(3-(*tert*-Butylperoxy)-4-iodobutyl)benzene (2m)

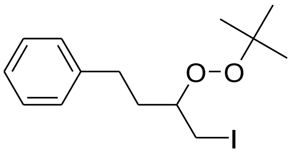

Yield: 80%; Pale yellow oil; ^{1}H NMR ($CDCl_3$, 400 Hz) δ 7.29 (d, J = 7.2 Hz, 2H), 7.21 (m, 3H), 2.36 (m, 1H), 3.83 (m, 1H), 3.48 (m, 1H), 3.36 (m, 1H), 2.74 (m, 2H), 1.98 (m, 2H), 1.28 (s, 9H); ^{13}C NMR ($CDCl_3$, 100 Hz) δ 141.3, 128.4, 128.3, 126.0, 81.1, 80.4, 34.3, 31.6, 26.6, 8.92; HRMS (ESI): calcd for $C_{14}H_{21}INaO_2$: [M+Na$^+$] 371.0478, found 371.0467.

3-(*tert*-Butylperoxy)-3-ethyl-2-iodopentane (2n)

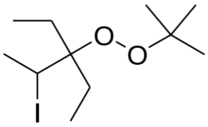

Yield: 81%; Orange oil; ^{1}H NMR ($CDCl_3$, 400 Hz) δ 4.66 (q, J = 7.6 Hz, 1H), 1.96 (s, 3H), 1.78 (m, 4H), 1.23 (s, 9H), 0.95 (m, 6H); ^{13}C NMR ($CDCl_3$, 100 Hz) δ 83.6, 78.7, 35.9, 27.1, 26.7, 26.2, 23.9, 8.69, 8.57; HRMS (ESI): calcd for $C_{11}H_{23}INaO_2$: [M+Na$^+$] 337.0635, found 337.0639.

2-(*tert*-Butylperoxy)-1-iodooctane (2o)

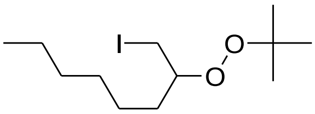

Yield: 70%; Orange oil; ^{1}H NMR ($CDCl_3$, 400 Hz) δ 3.77 (m, 1H), 3.47 (dd, *J* = 10.0 Hz, *J* = 3.2 Hz, 1H), 3.32 (dd, *J* = 10.0 Hz, *J* = 3.2 Hz, 1H), 1.61 (m, 3H), 1.32 (m, 7H), 1.25 (s, 9H), 0.89 (t, *J* = 7.6 Hz, 3H); ^{13}C NMR ($CDCl_3$, 100 Hz) δ 81.9, 80.3, 32.5, 31.6, 29.1, 26.5, 25.4, 22.5, 14.0, 9.34; HRMS (ESI): calcd for $C_{12}H_{25}INaO_2$: [M+Na$^+$] 351.0791, found 351.0782.

1-(2-(*tert*-Butylperoxy)-3-iodopropyl)benzene (2p)

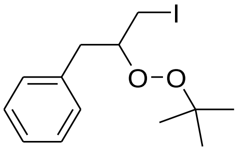

Yield: 78%; Orange oil; ^{1}H NMR ($CDCl_3$, 400 Hz) δ 7.31 (m, 5H), 4.04 (m, 1H), 3.43 (dd, *J* = 10.0 Hz, *J* = 3.2 Hz, 1H), 3.31 (dd, *J* = 10.0 Hz, *J* = 3.2 Hz, 1H), 3.02 (dd, *J* = 14.0 Hz, *J* = 6.8 Hz, 1H), 2.92 (dd, *J* = 14.0 Hz, *J* = 6.8 Hz, 1H), 1.20 (s, 9H); ^{13}C NMR ($CDCl_3$, 100 Hz) δ 137.2, 129.5, 128.3, 126.6, 83.0, 80.7, 38.5, 26.4, 8.13; HRMS (ESI): calcd for $C_{13}H_{19}INaO_2$: [M+Na$^+$] 357.0322, found 357.0329.

1-(1-(*tert*-Butylperoxy)-2-iodoethyl)-4-benzylbenzene (2q)

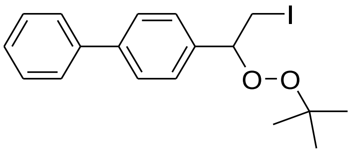

Yield: 83%; Colorless oil; ^{1}H NMR ($CDCl_3$, 400 Hz) δ 7.63 (d, *J* = 7.6 Hz, 4H), 7.46 (m, 4H), 7.38 (t, *J* = 7.6 Hz, 1H), 5.10 (t, *J* = 6.8 Hz, 1H), 3.70 (dd, *J* = 10.0 Hz, *J* = 6.4 Hz, 1H), 3.46 (dd, *J* = 10.0 Hz, *J* = 6.4 Hz, 1H), 1.33 (s, 9H); ^{13}C NMR ($CDCl_3$, 100 Hz) δ 141.5, 140.6, 137.2, 128.8, 127.5, 127.4, 127.2, 127.1, 85.1, 81.2, 26.5, 6.27; HRMS (ESI): calcd for $C_{18}H_{21}INaO_2$: [M+Na$^+$] 419.0478, found 419.0486.

1-*tert*-Butyl-4-(1-(*tert*-butylperoxy)-2-iodoethyl)benzene (2r)

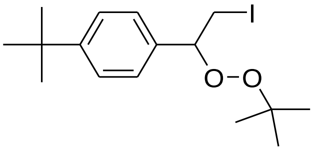

Yield: 82%; Orange oil; ^{1}H NMR ($CDCl_3$, 400 Hz) δ 7.42 (d, *J* = 7.6 Hz, 2H), 7.31 (d, *J* = 7.6 Hz, 2H), 5.05 (t, *J* = 6.8 Hz, 1H), 3.68 (dd, *J* = 10.0 Hz, *J* = 3.6 Hz, 1H), 3.43 (dd, *J* = 10.0 Hz, *J* = 3.6 Hz, 1H), 1.37 (s, 9H), 1.32 (s, 9H); ^{13}C NMR ($CDCl_3$, 100 Hz) δ 151.4, 135.1, 126.8, 125.4, 85.2, 81.0, 34.6, 31.4, 26.6, 6.58; HRMS (ESI): calcd for $C_{16}H_{25}INaO_2$: [M+Na$^+$] 399.0791, found 399.0795.

1-(1-(*tert*-Butylperoxy)-2-iodoethyl)-4-(chloromethyl)benzene (2s)

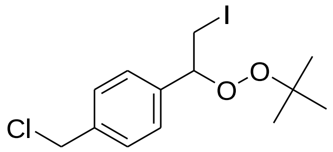

Yield: 76%; Orange oil; ^{1}H NMR ($CDCl_3$, 400 Hz) δ 7.41 (d, *J* = 7.6 Hz, 2H), 7.36 (d, *J* = 7.6 Hz, 2H), 5.03 (t, *J* = 6.8 Hz, 1H), 4.59 (s, 2H), 3.68 (dd, *J* = 10.0 Hz, *J* = 6.0 Hz, 1H), 3.43 (dd, *J* = 10.0 Hz, *J* = 6.0 Hz, 1H), 1.29 (s, 9H); ^{13}C NMR ($CDCl_3$, 100 Hz) δ 138.6, 137.7, 128.7, 127.4, 84.8, 81.2, 45.9, 26.5, 6.14; HRMS (ESI): calcd for $C_{13}H_{18}ClINaO_2$: [M+Na$^+$] 390.9932, found 390.9939.

1-(*tert*-Butylperoxy)-2-iodo-1-methylcyclopentane (2t)

Yield: 80%; Pale red oil; ^{1}H NMR ($CDCl_3$, 400 Hz) δ 4.66 (d, *J* = 6.0 Hz, 1H), 2.44 (m, 1H), 2.04 (m, 1H), 1.84 (m, 3H), 1.69 (m, 1H), 1.55 (s, 3H), 1.19 (s, 9H); ^{13}C NMR ($CDCl_3$, 100 Hz) δ 90.7, 78.7, 37.4, 36.6, 33.3, 26.6, 25.2, 22.5; HRMS (ESI): calcd for $C_{10}H_{19}INaO_2$: [M+Na$^+$] 321.0322, found 321.0336.

NMR spectra

1-(1-(*tert*-Butylperoxy)-2-iodoethyl)benzene (2a)

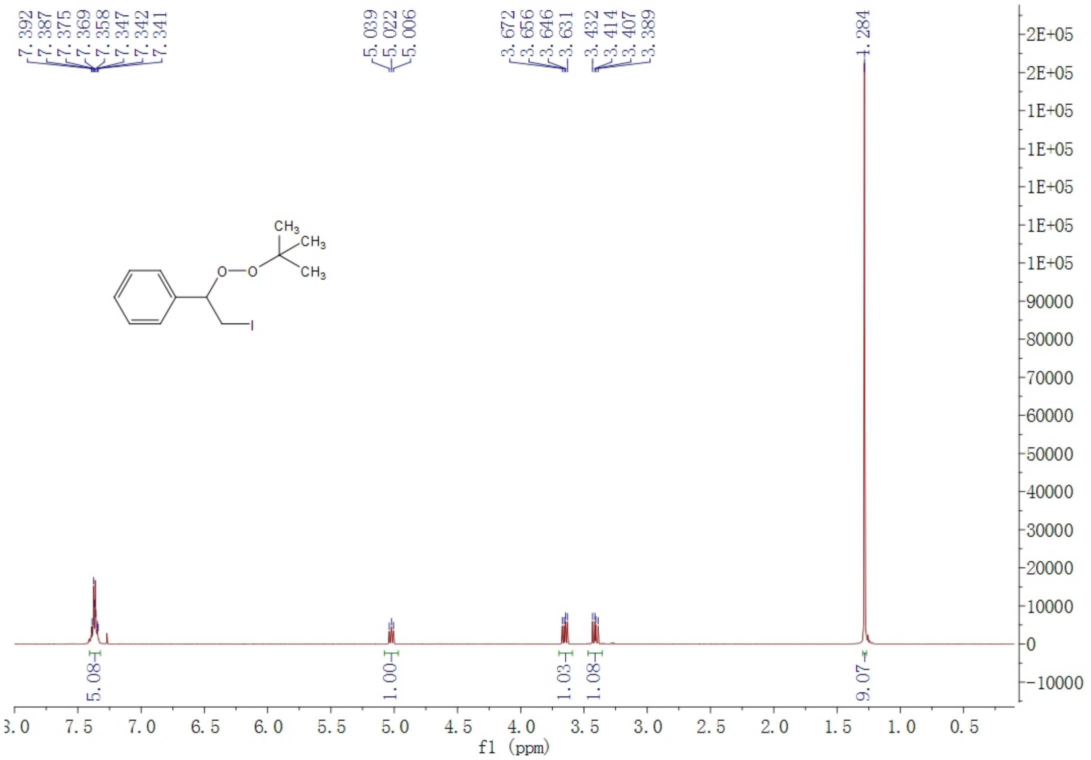

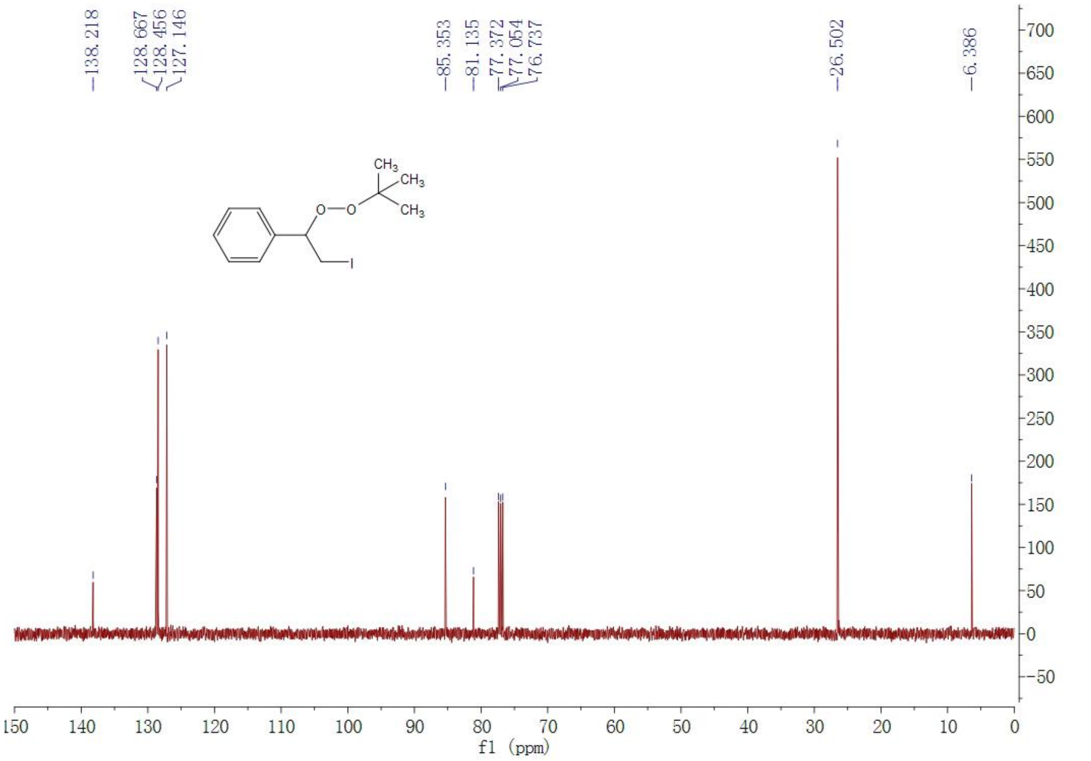

1-(1-(*tert*-Butylperoxy)-2-iodoethyl)-4-methylbenzene (2b)

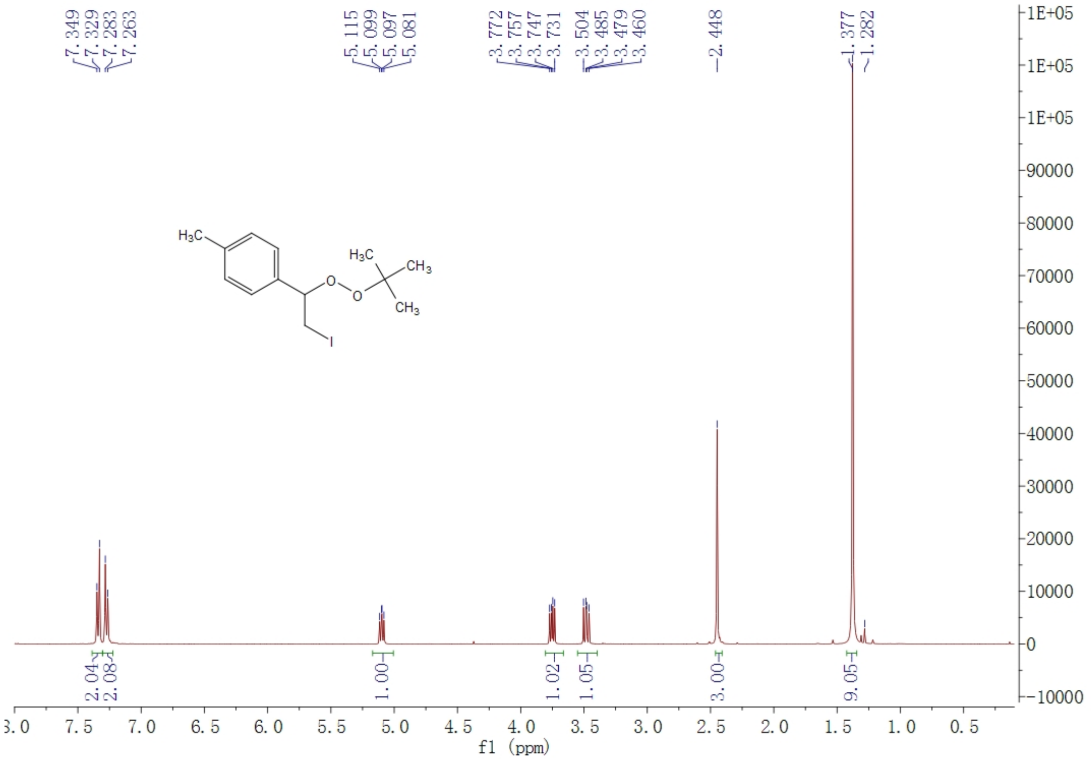

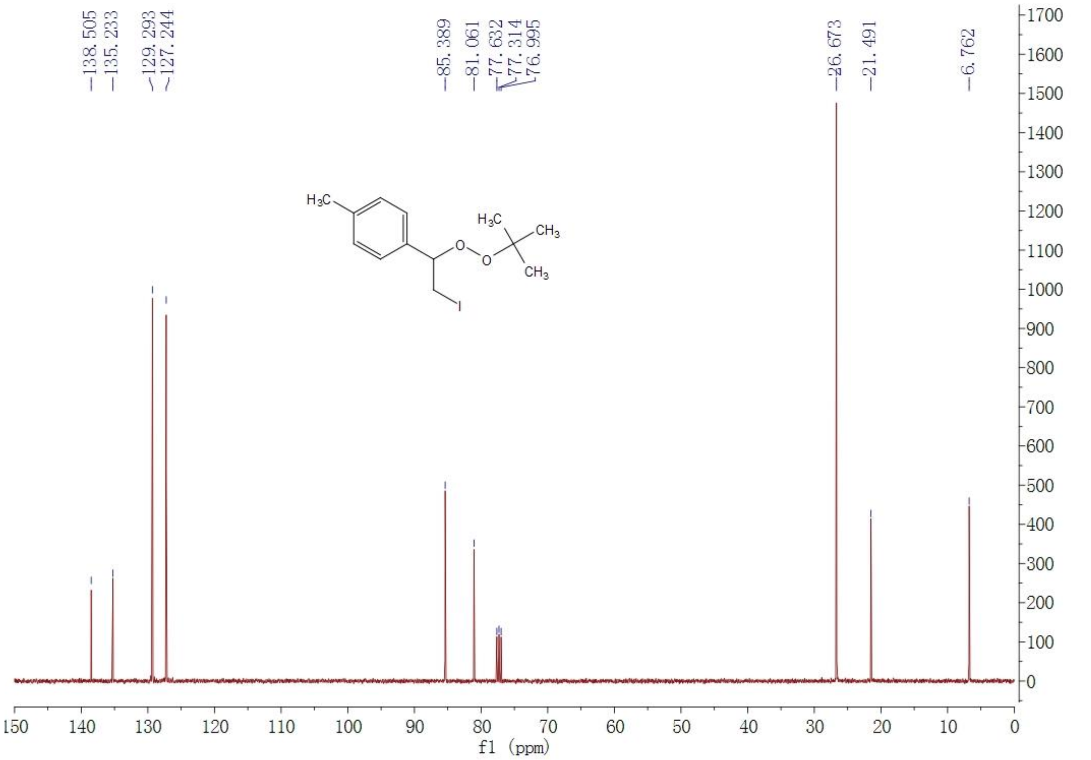

2-(1-(*tert*-Butylperoxy)-2-iodoethyl)-1,4-dimethylbenzene (2c)

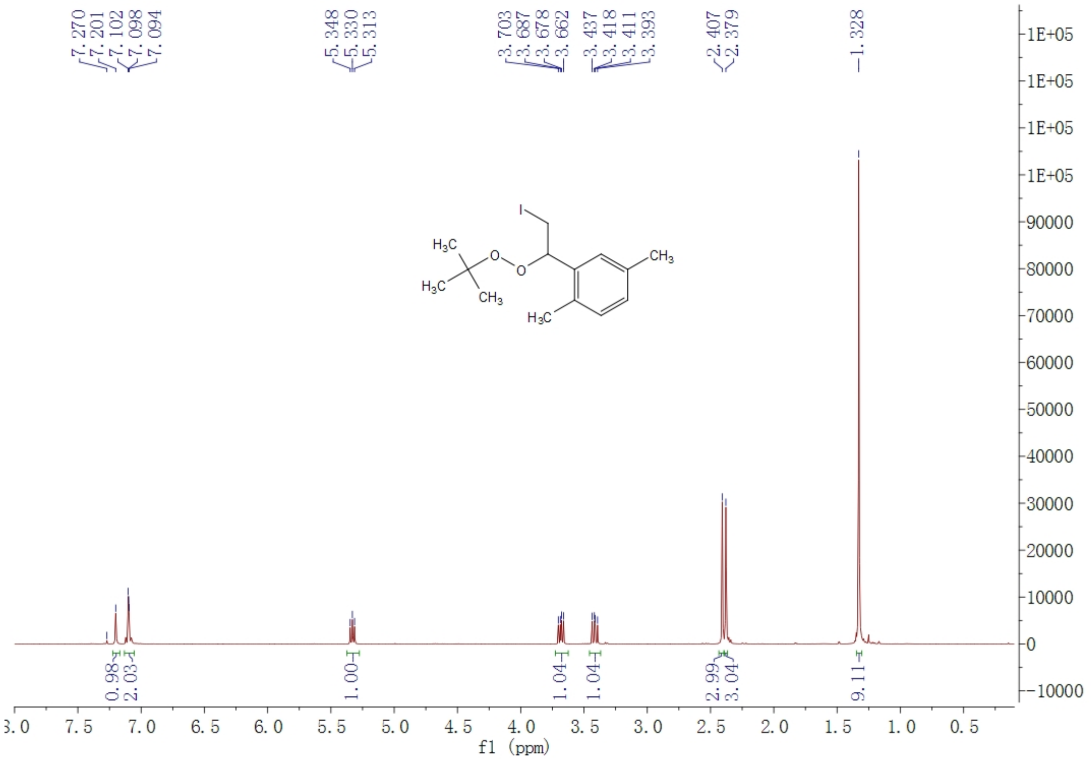

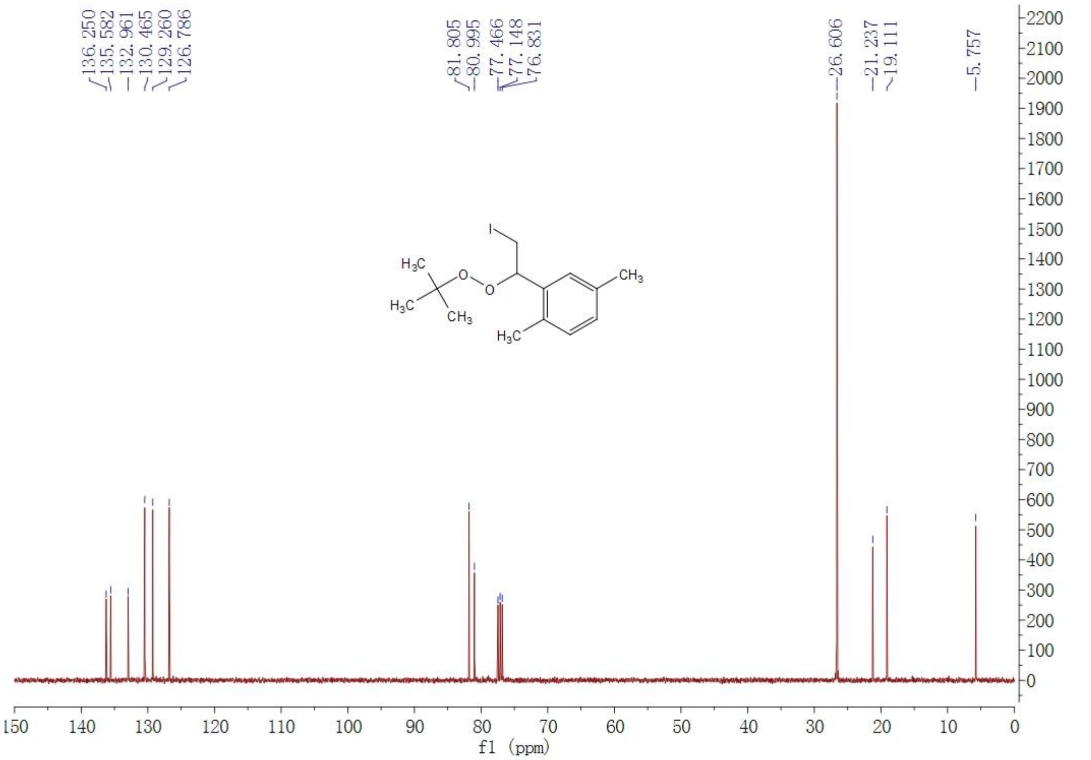

1-(1-(*tert*-Butylperoxy)-2-iodoethyl)-4-methoxybenzene (2d)

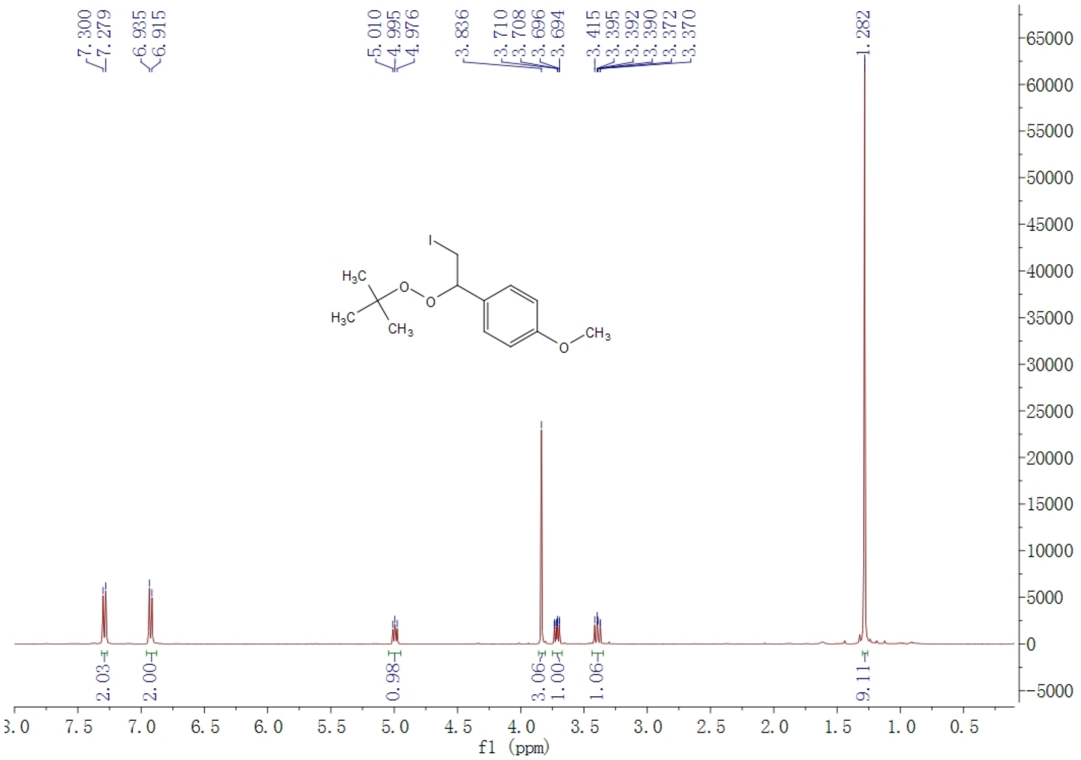

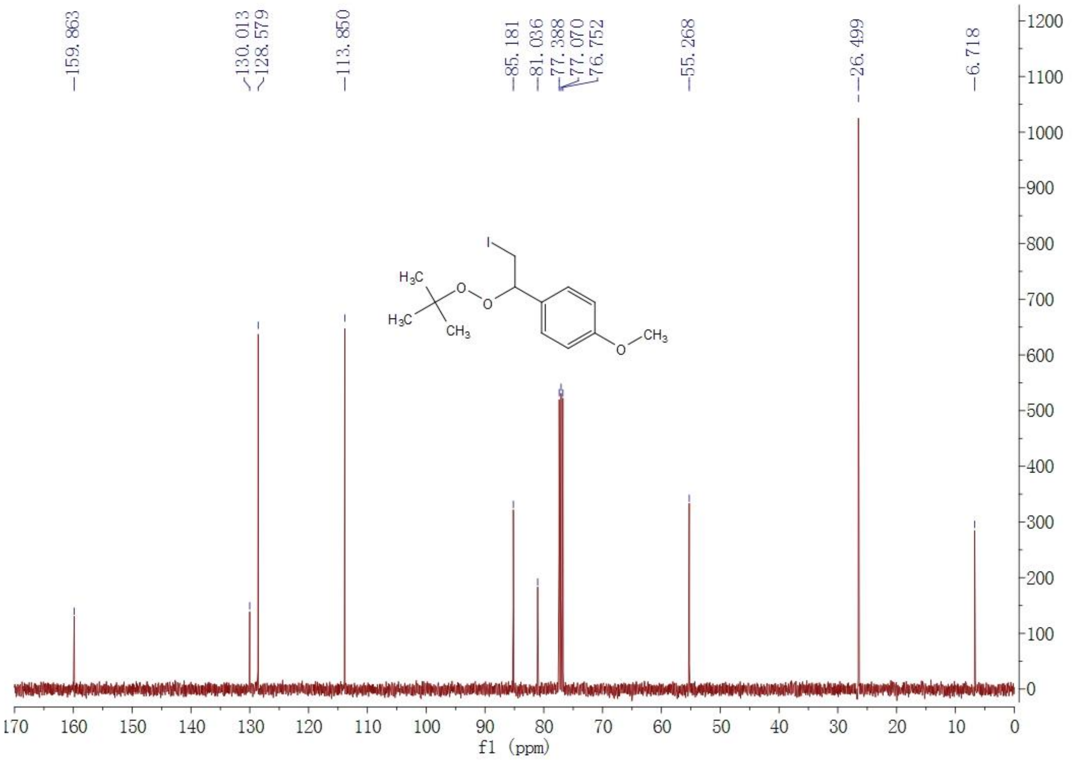

1-(1-(*tert*-Butylperoxy)-2-iodoethyl)-4-chlorobenzene (2e)

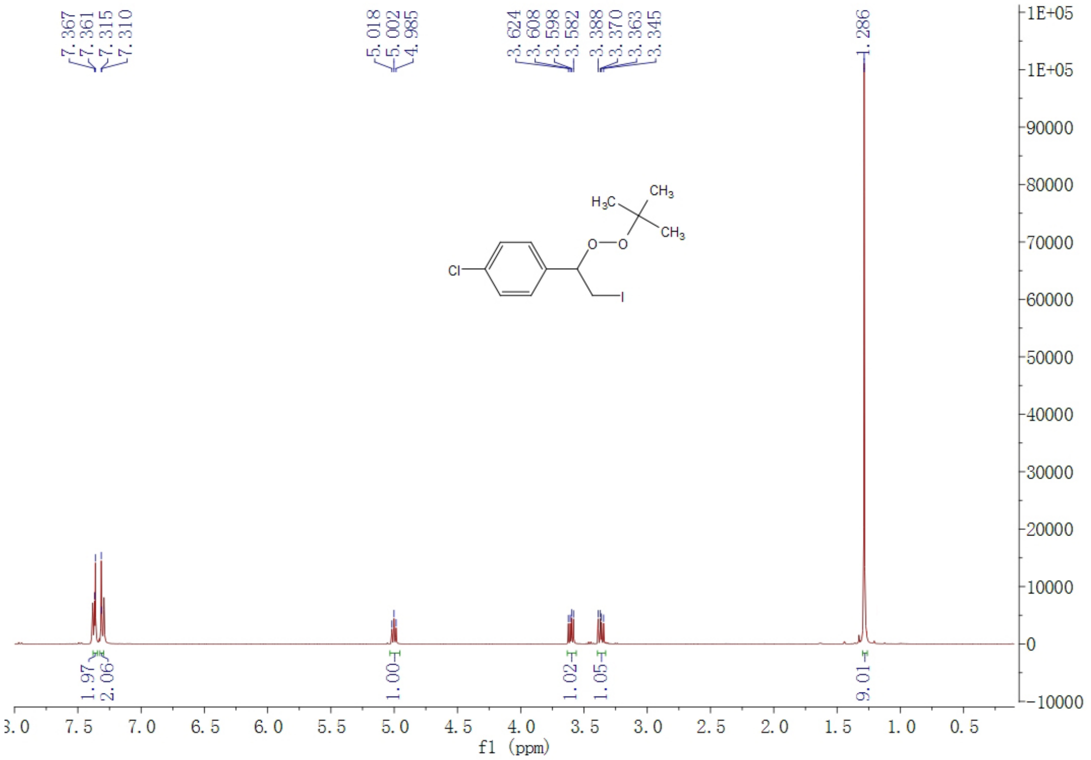

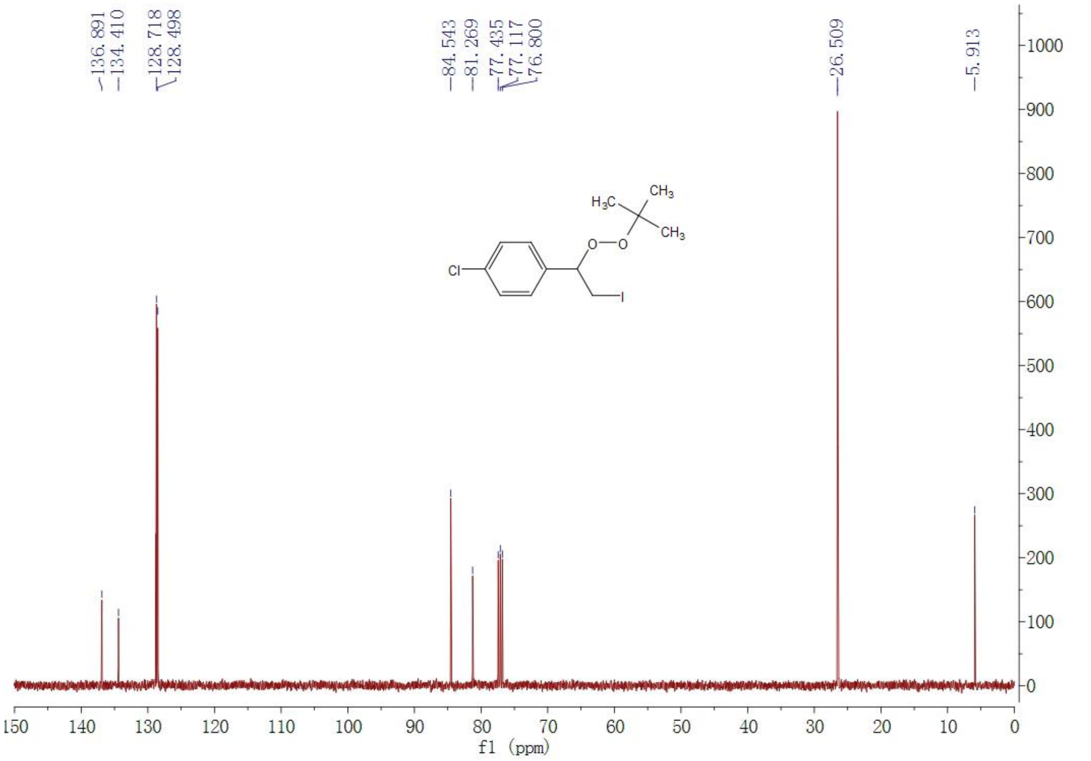

1-(1-(*tert*-Butylperoxy)-2-iodoethyl)-2-chlorobenzene (2g)

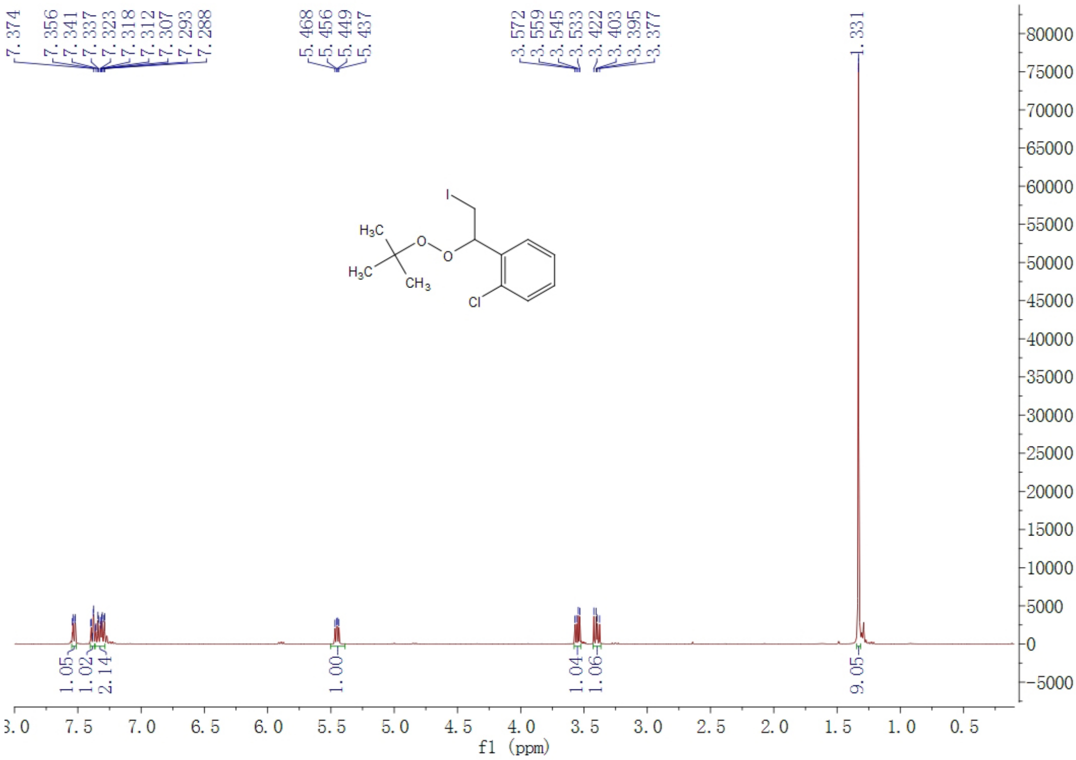

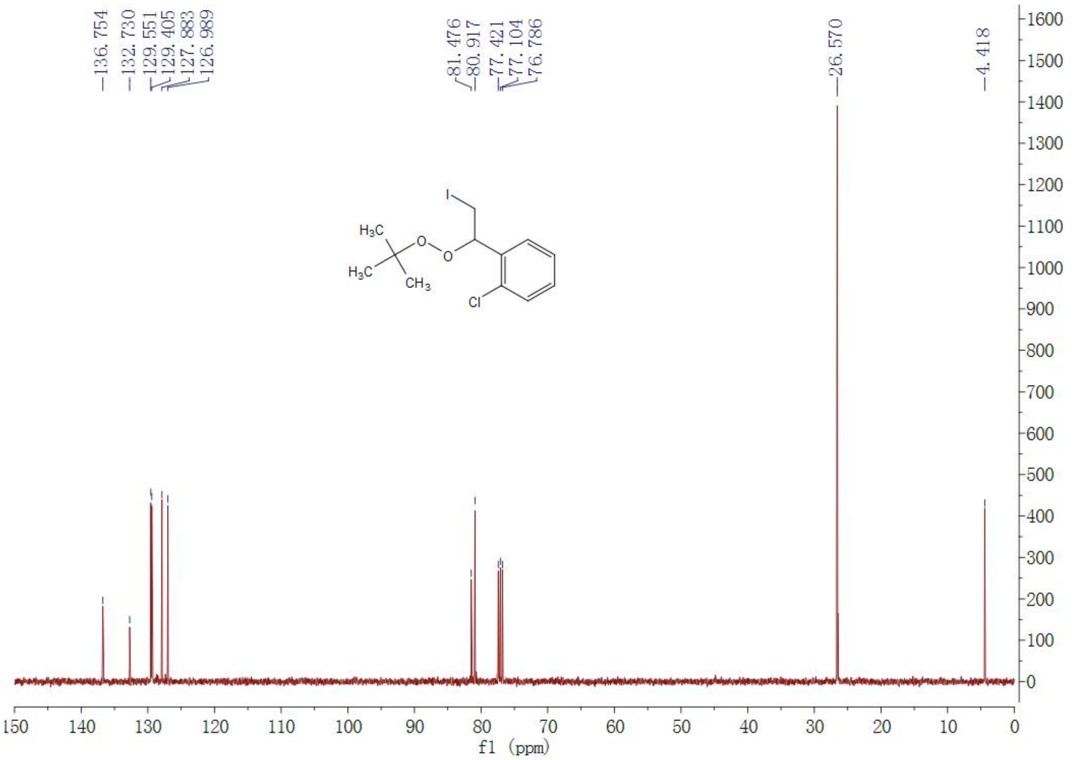

1-(1-(*tert*-Butylperoxy)-2-iodoethyl)-4-fluorobenzene (2h)

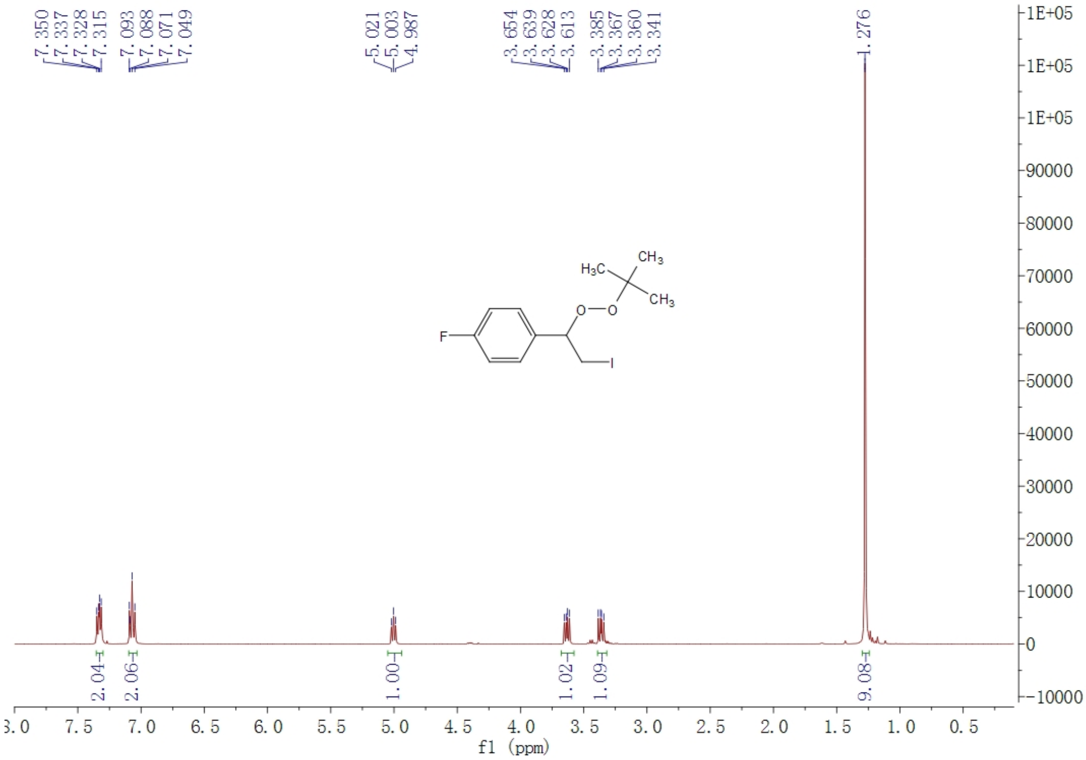

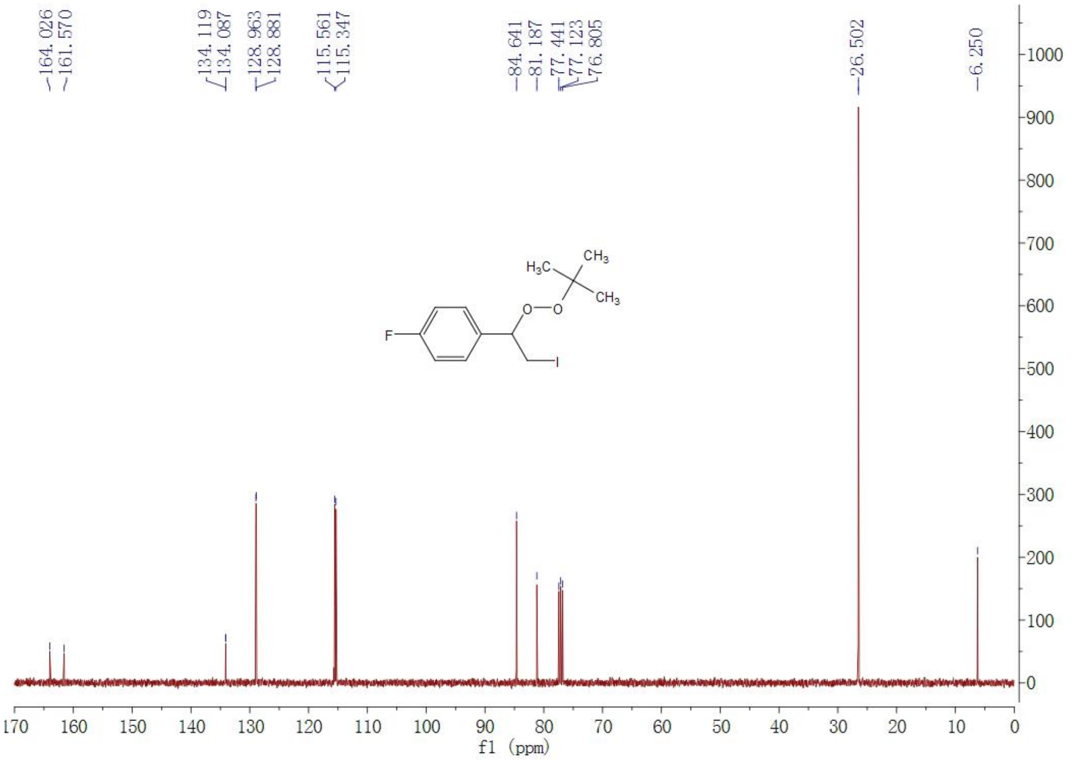

1-(1-(*tert*-Butylperoxy)-2-iodoethyl)-3-fluorobenzene (2i)

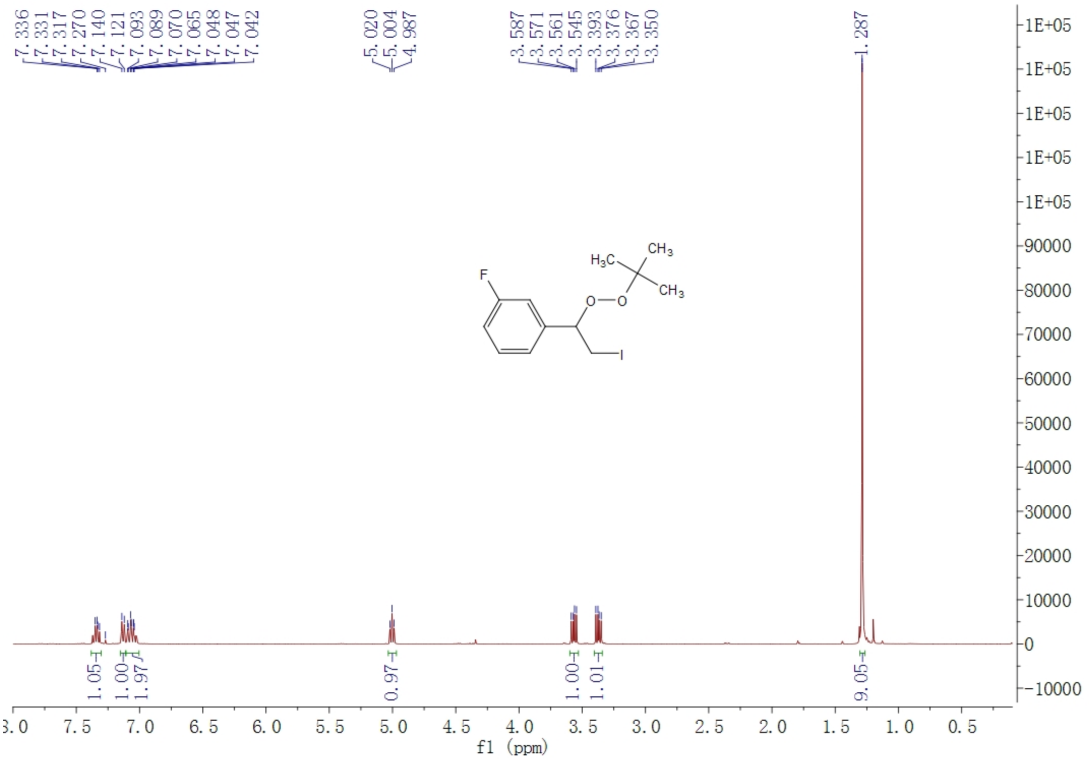

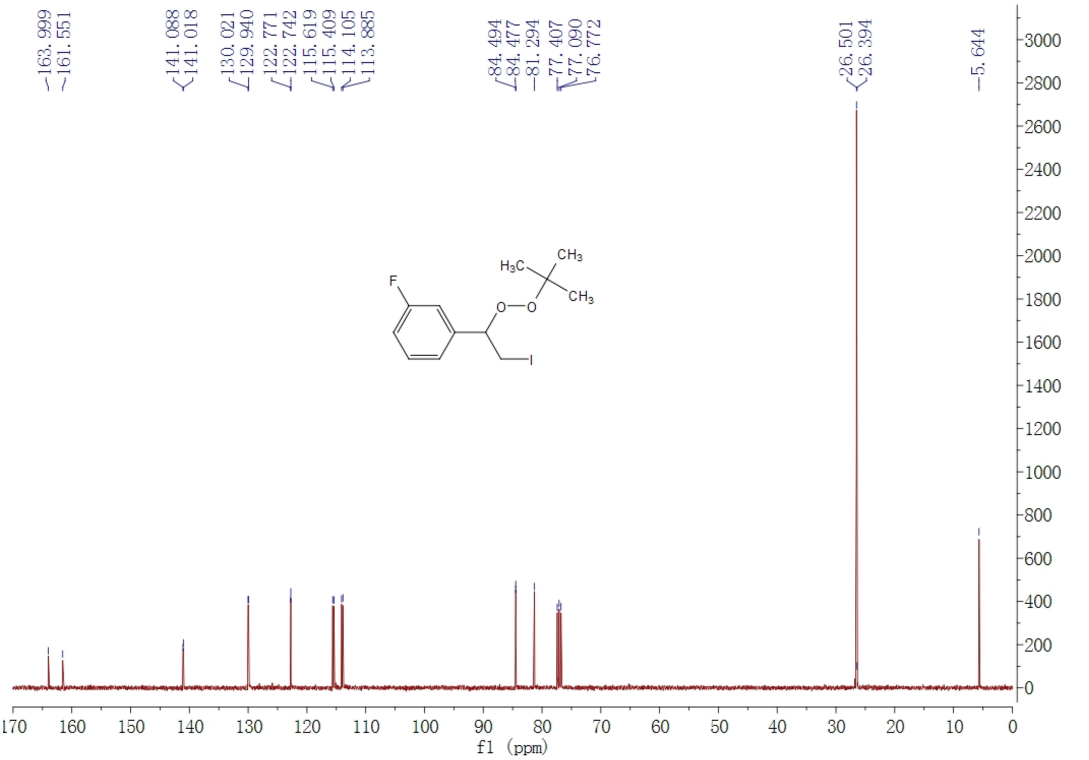

1-(2-(*tert*-Butylperoxy)-1-iodopropan-2-yl)-4-methylbenzene (2j)

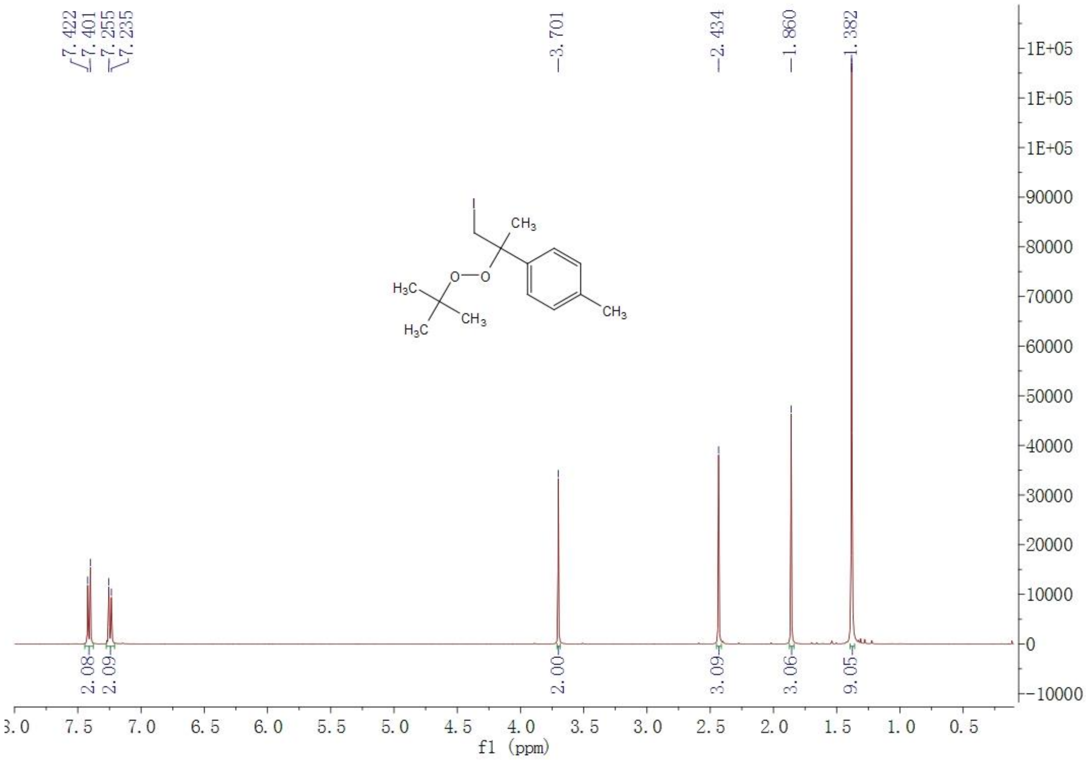

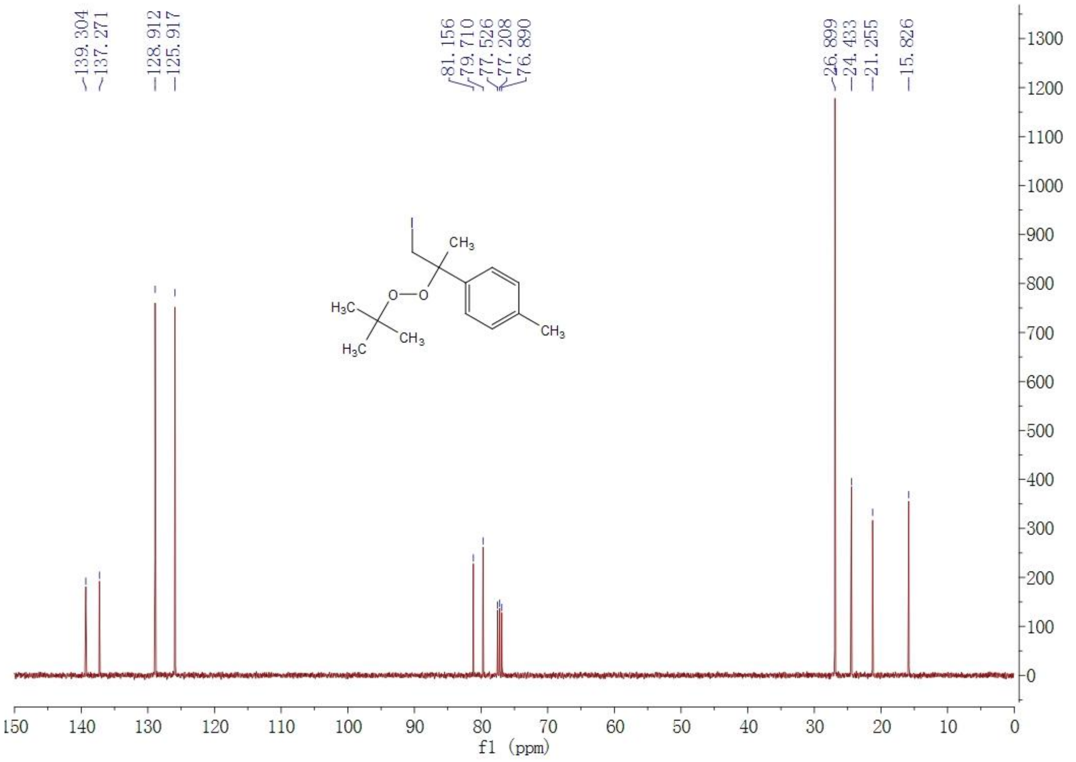

1-(2-(*tert*-Butylperoxy)-1-iodopropan-2-yl)benzene (2k)

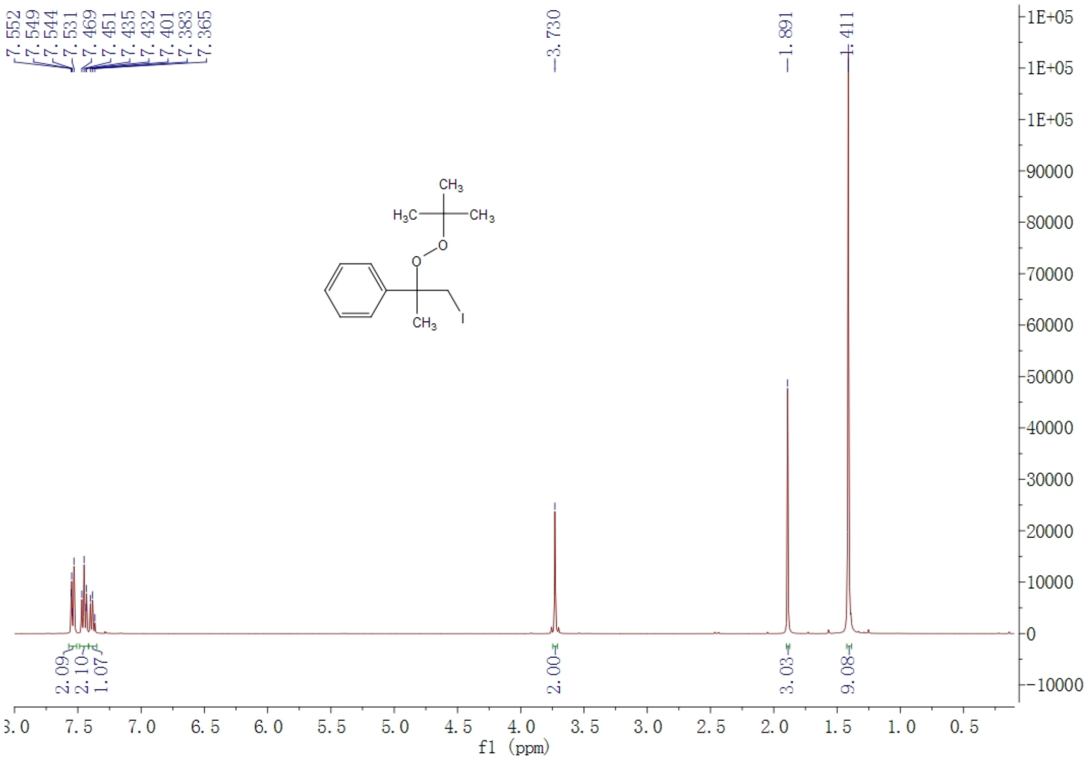

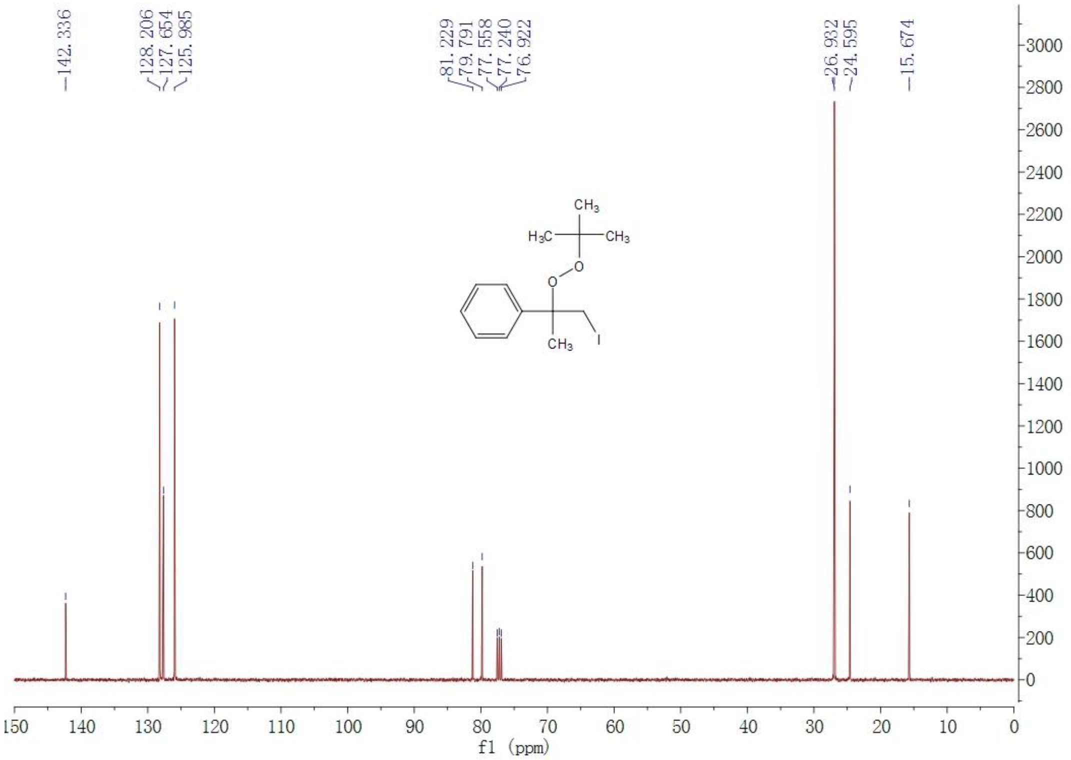

1-(*tert*-Butylperoxy)-2-iodo-1,1-diphenylethane (2l)

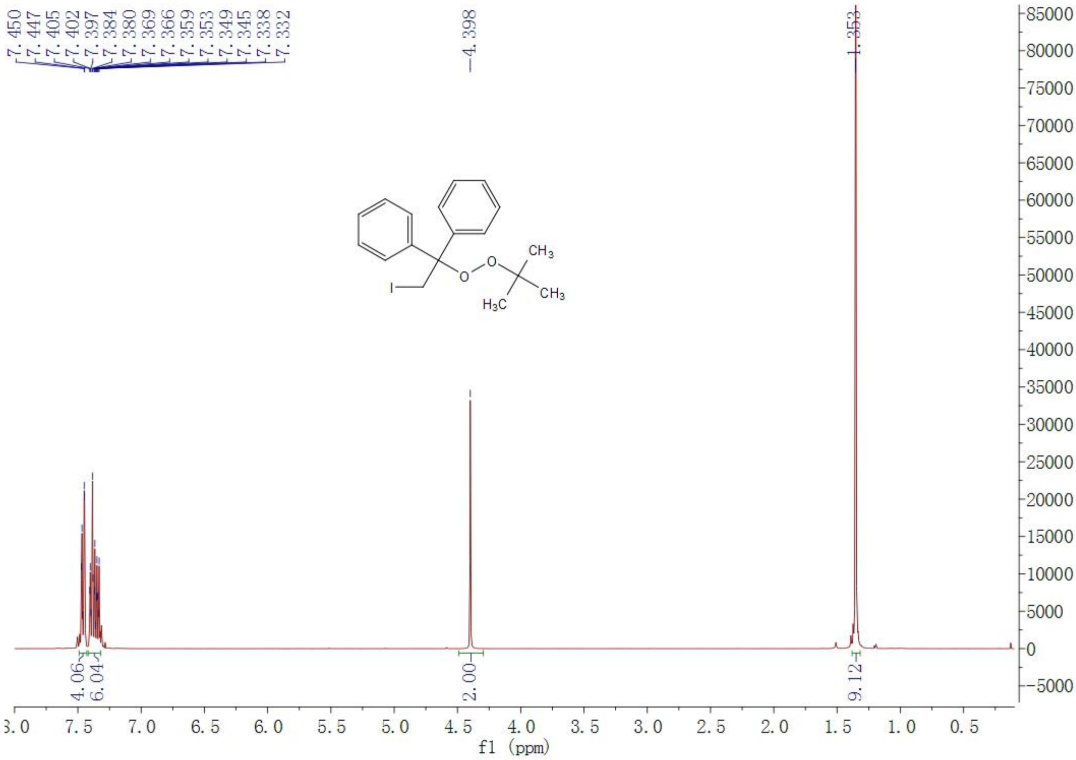

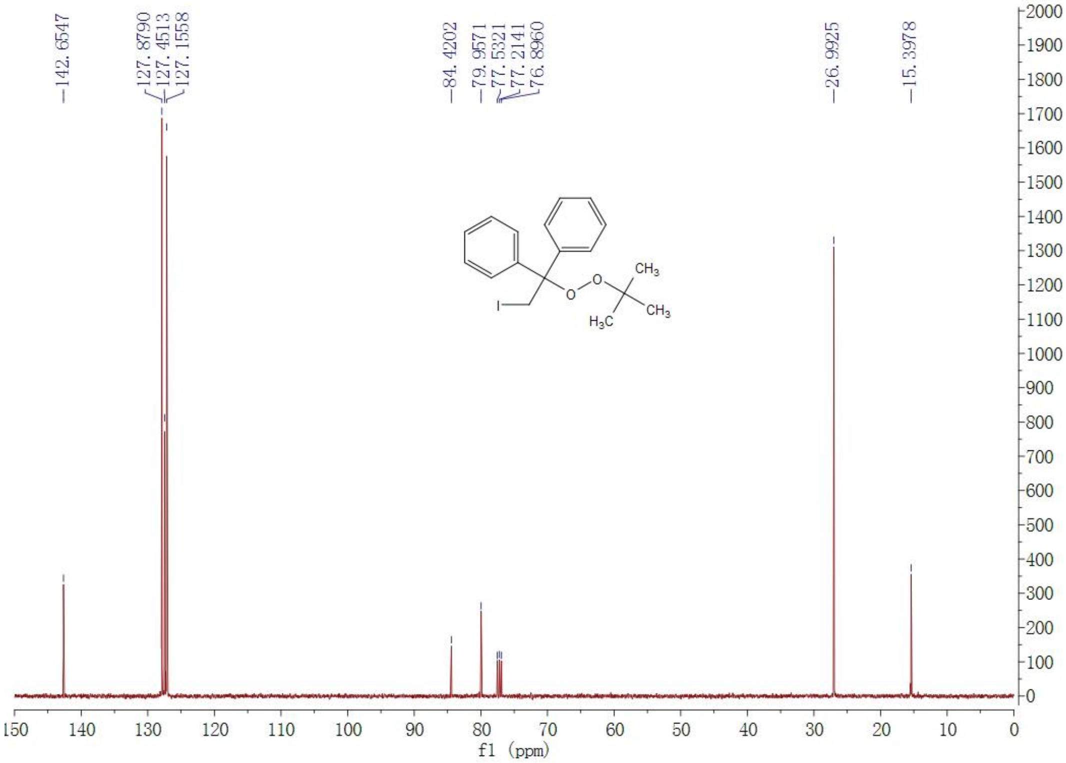

1-(3-(*Tert*-butylperoxy)-4-iodobutyl)benzene (2m)

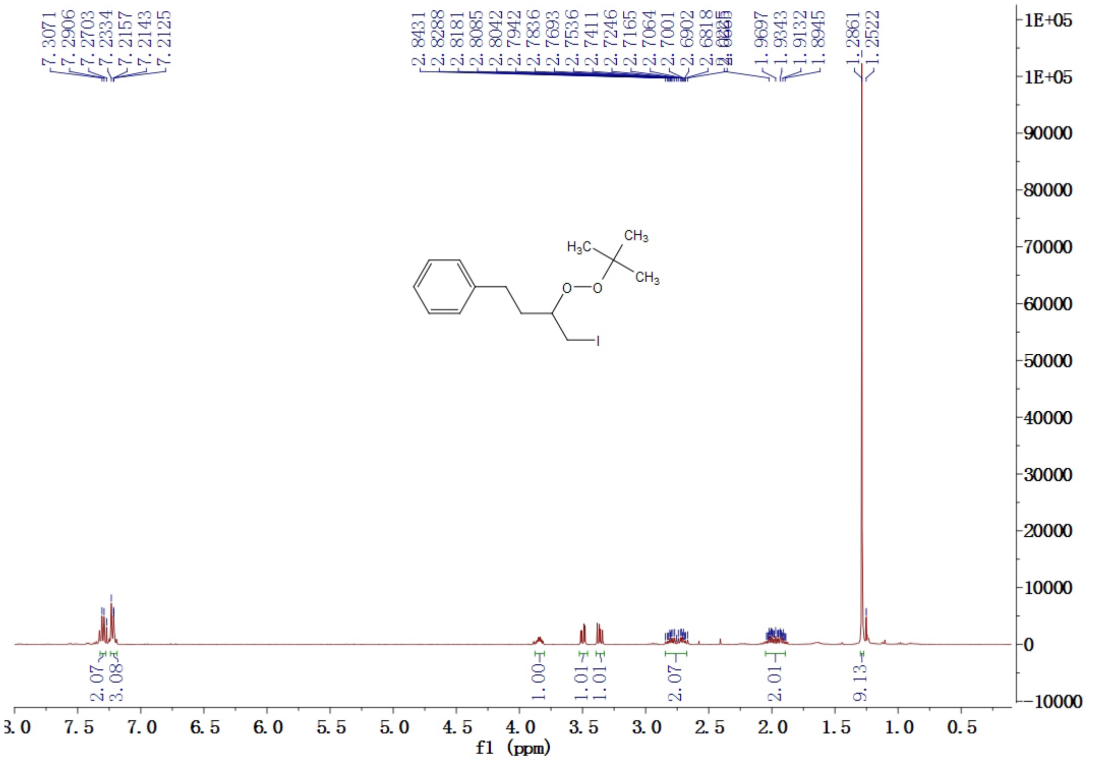

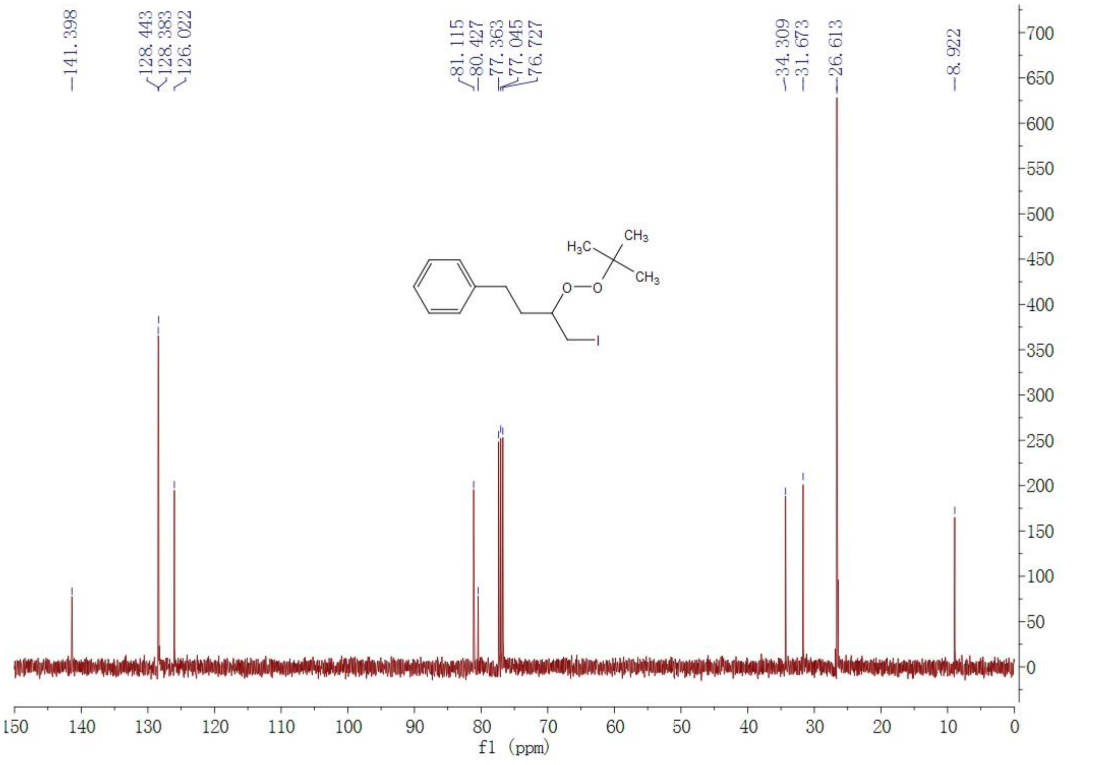

3-(*tert*-Butylperoxy)-3-ethyl-2-iodopentane (2n)

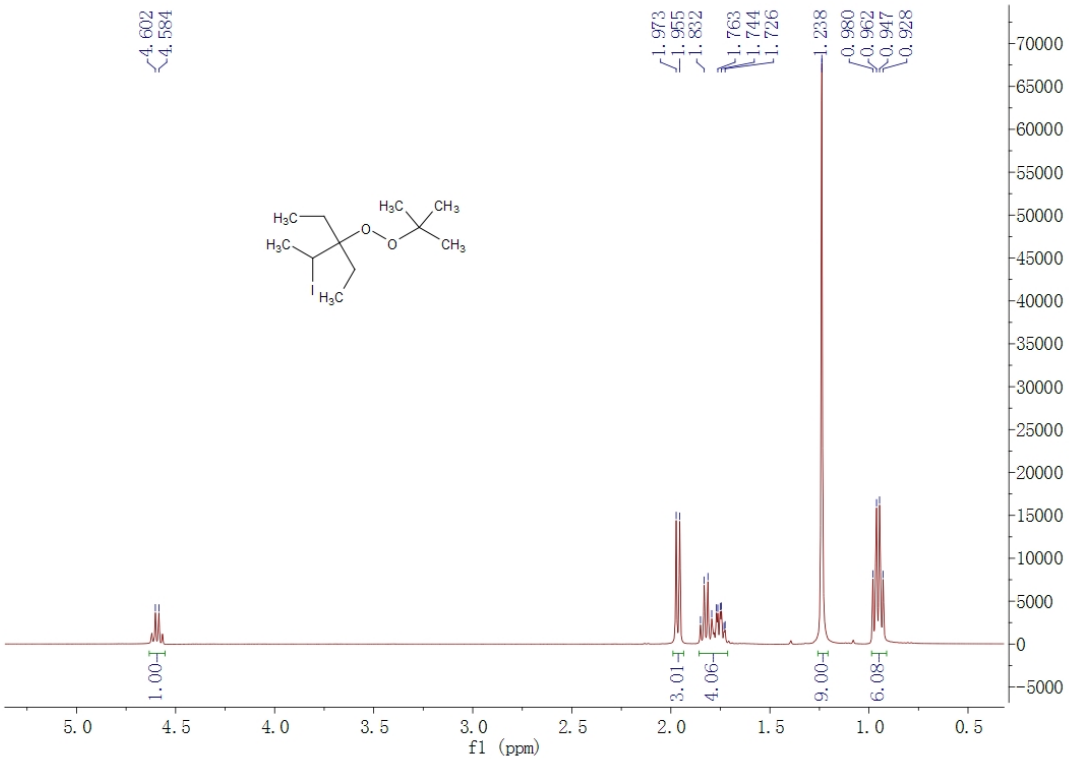

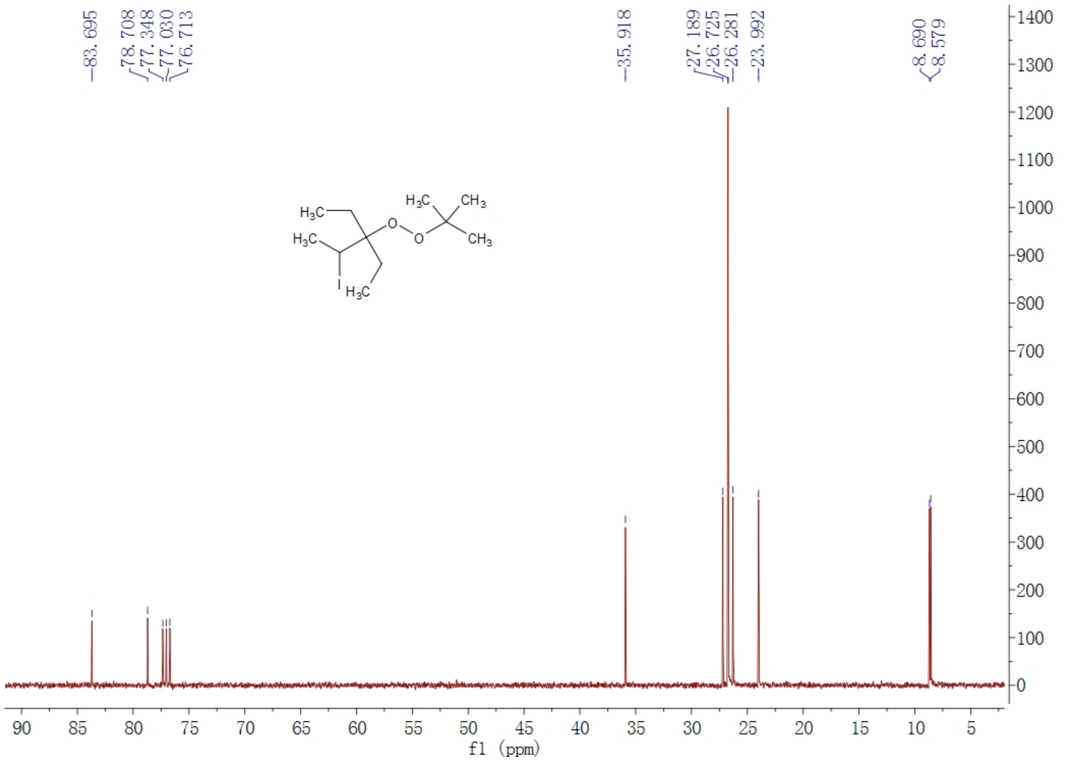

2-(*tert*-Butylperoxy)-1-iodooctane (2o)

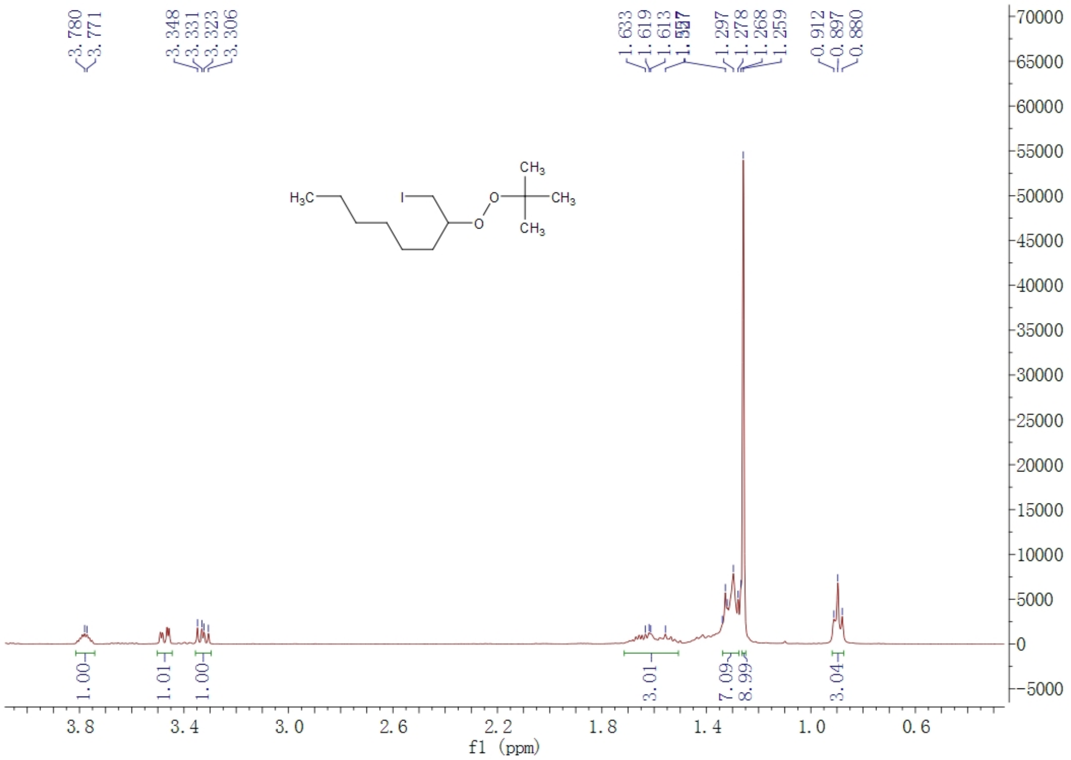

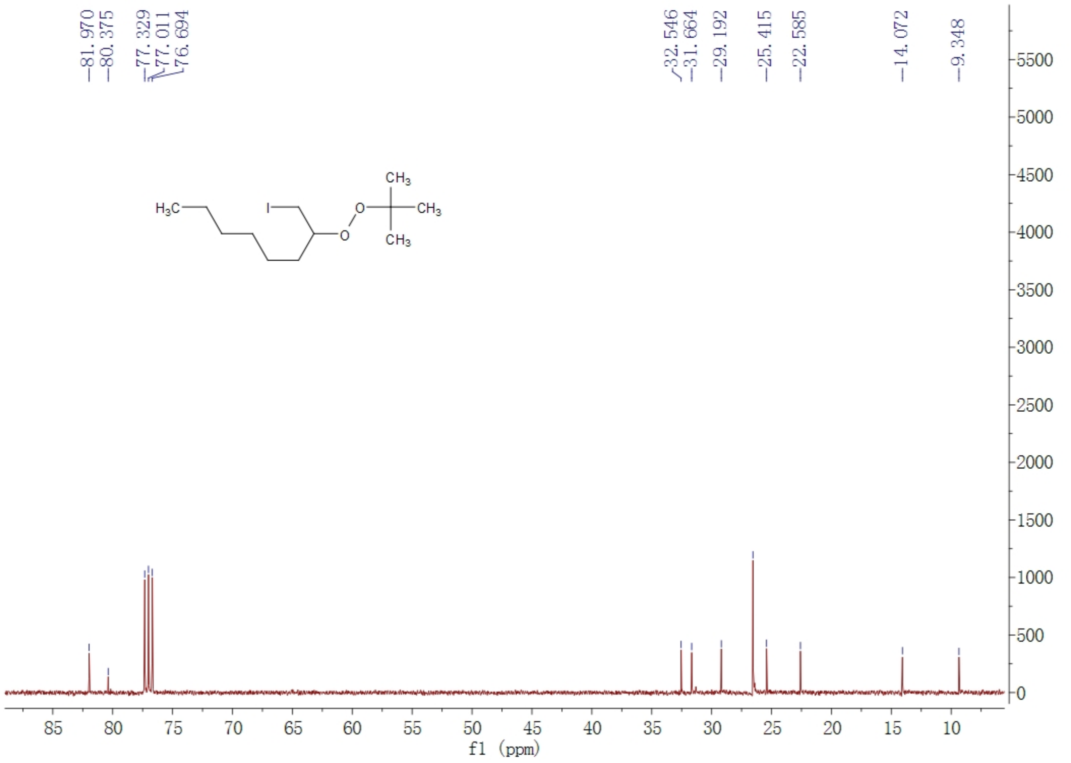

1-(2-(*tert*-Butylperoxy)-3-iodopropyl)benzene (2p)

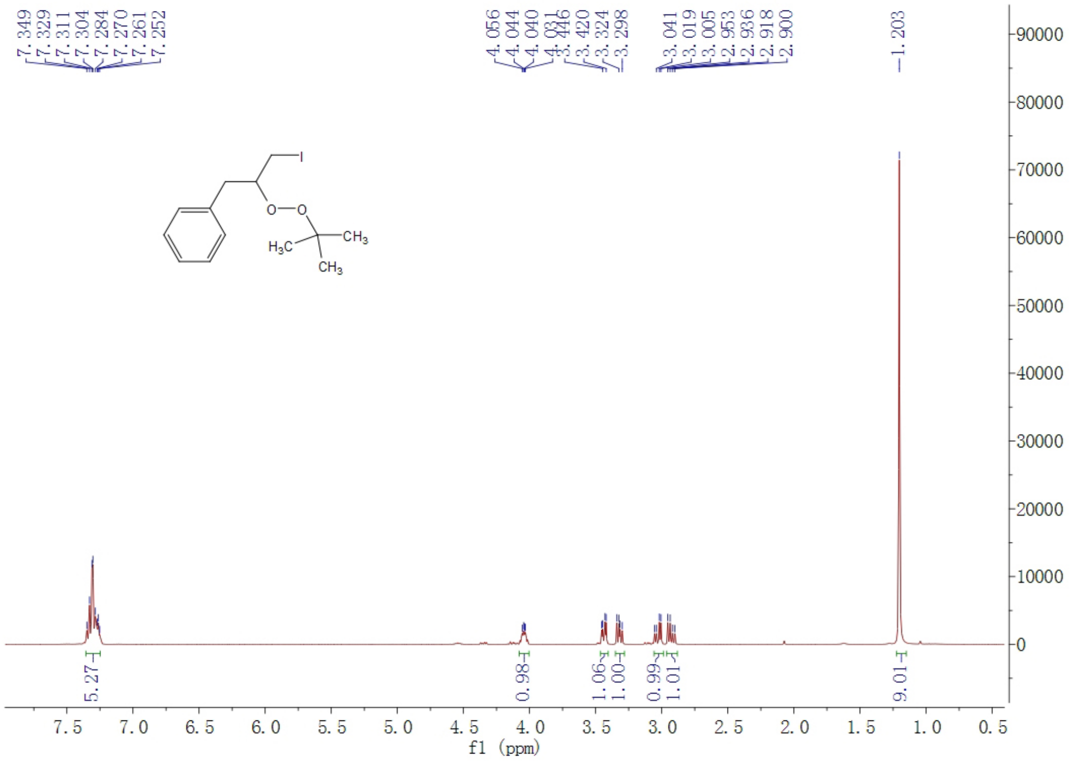

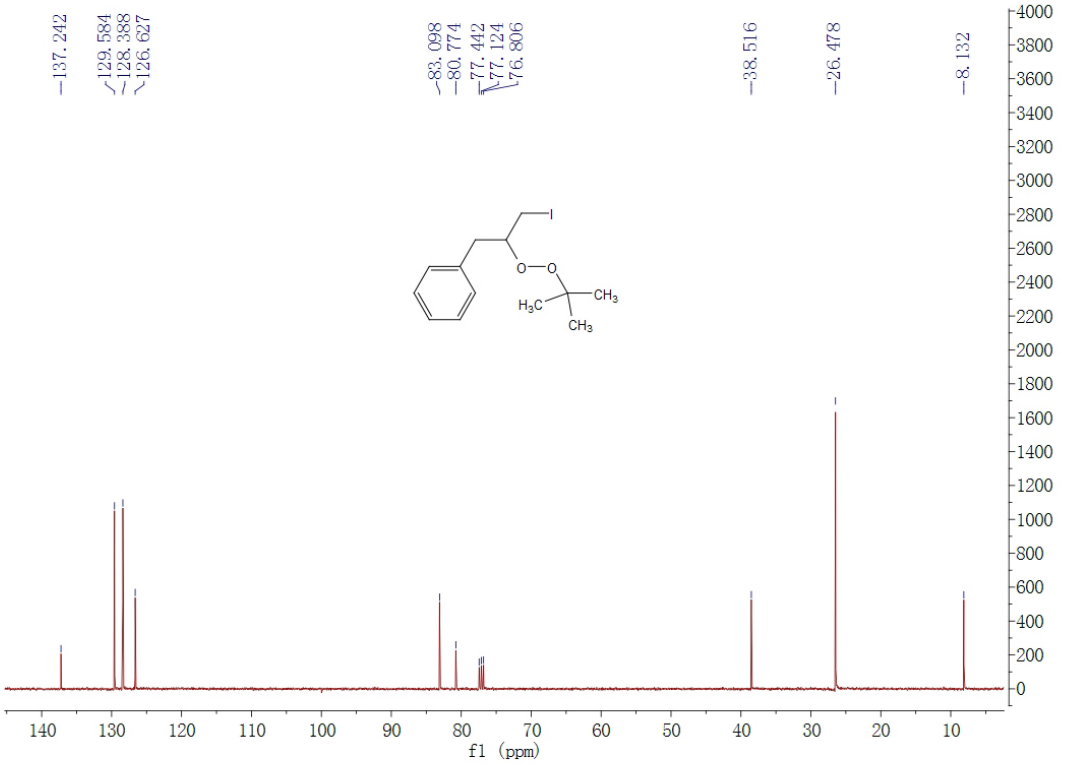

1-(1-(*tert*-Butylperoxy)-2-iodoethyl)-4-benzylbenzene (2q)

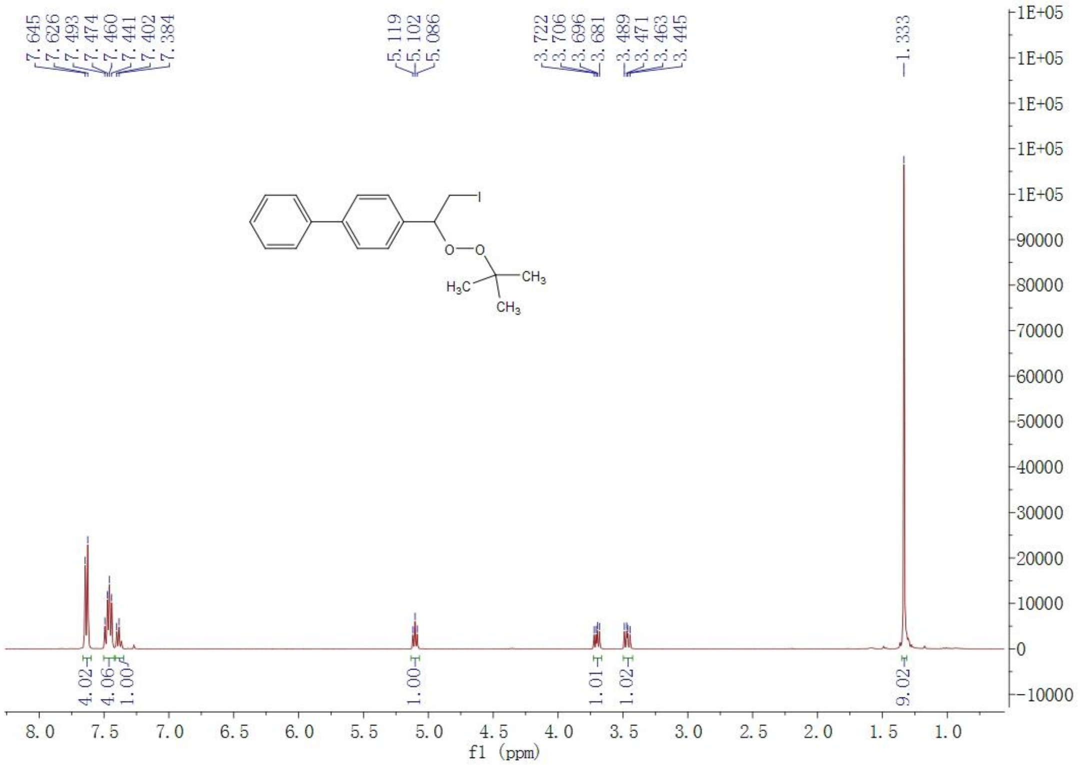

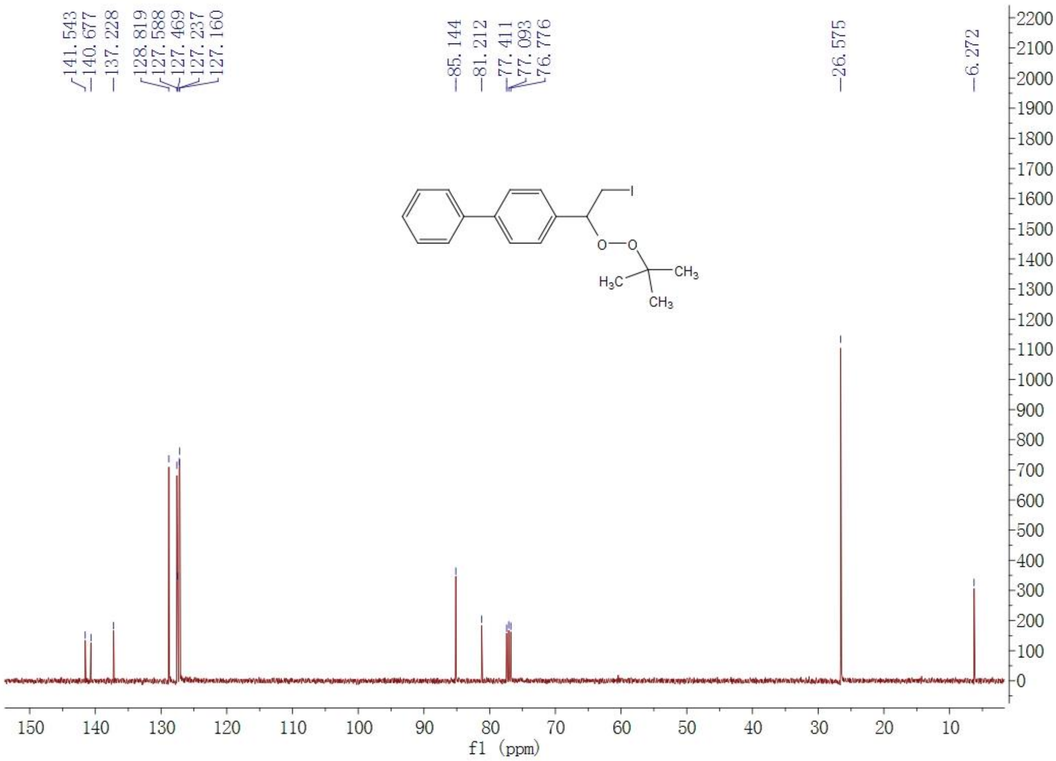

1-*tert*-Butyl-4-(1-(*tert*-butylperoxy)-2-iodoethyl)benzene (2r)

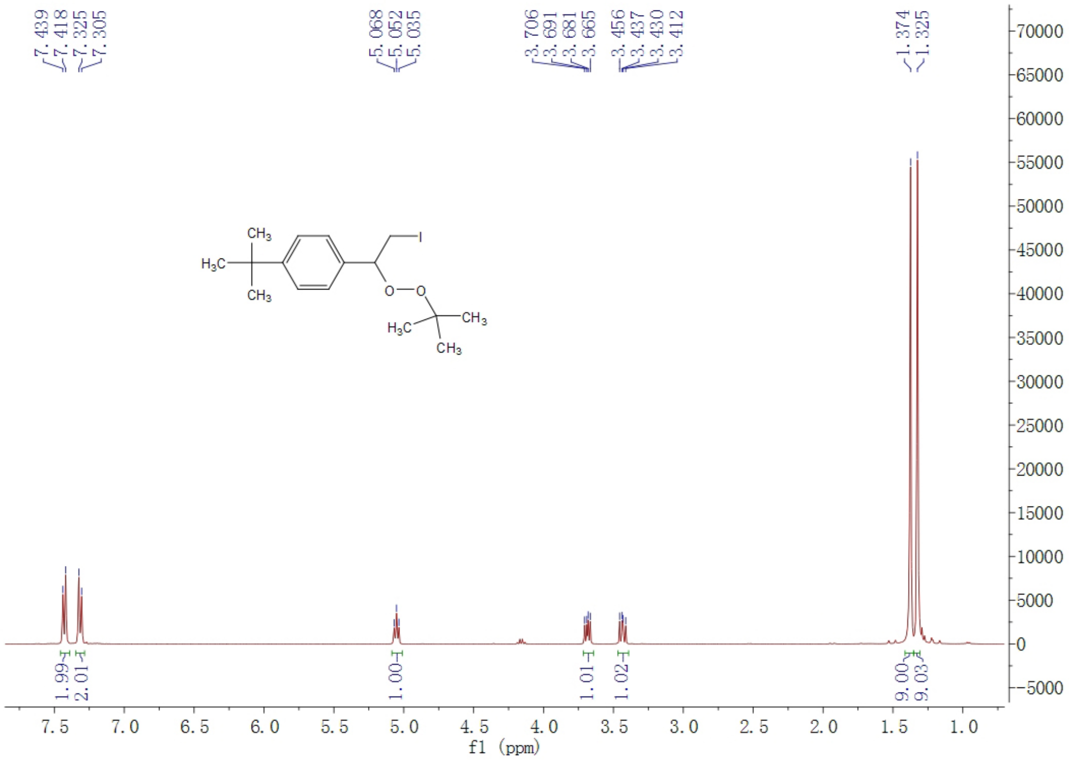

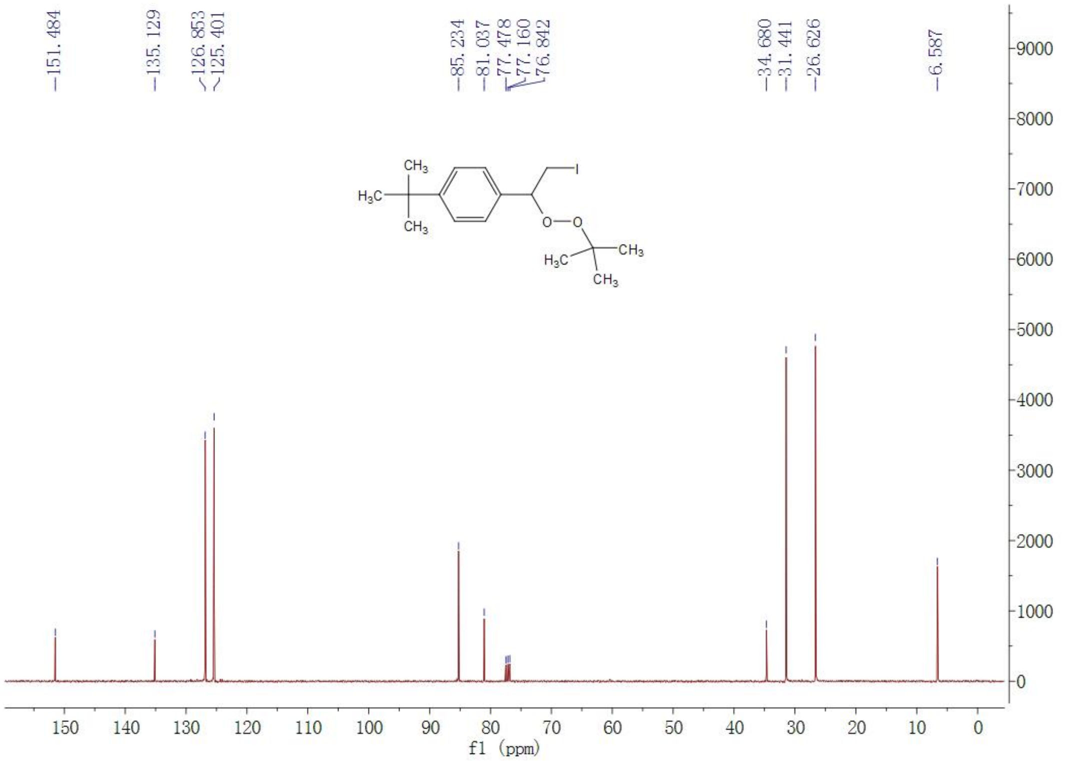

1-(1-(*tert*-Butylperoxy)-2-iodoethyl)-4-(chloromethyl)benzene (2s)

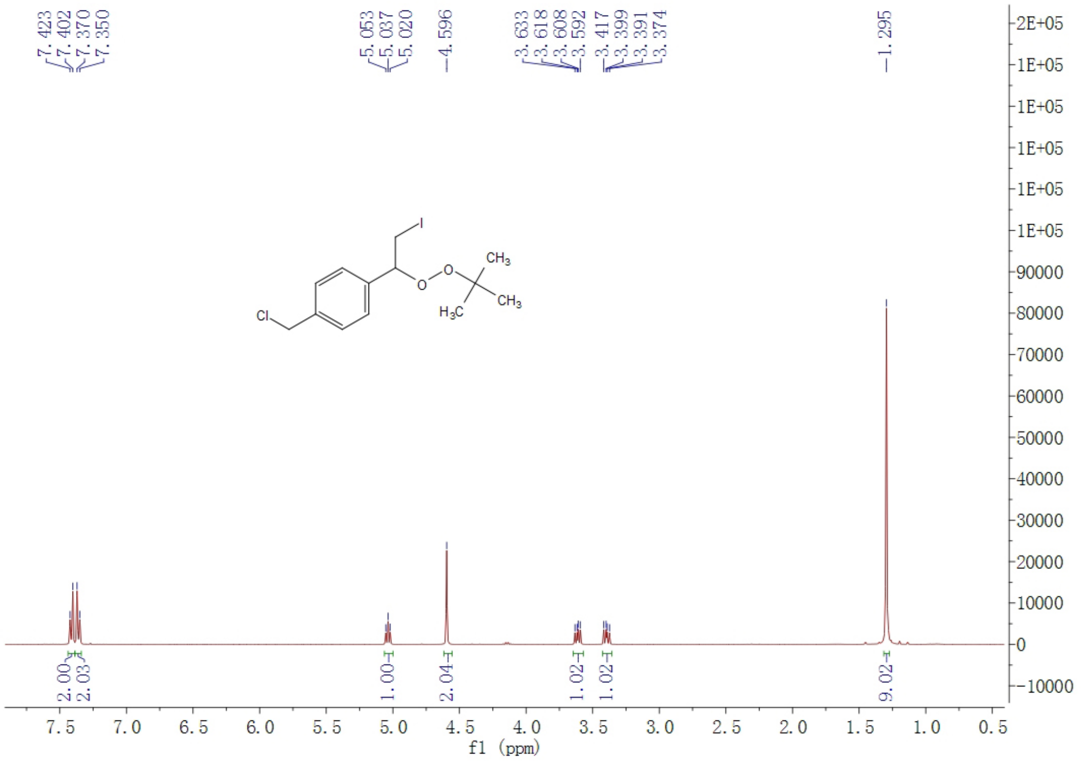

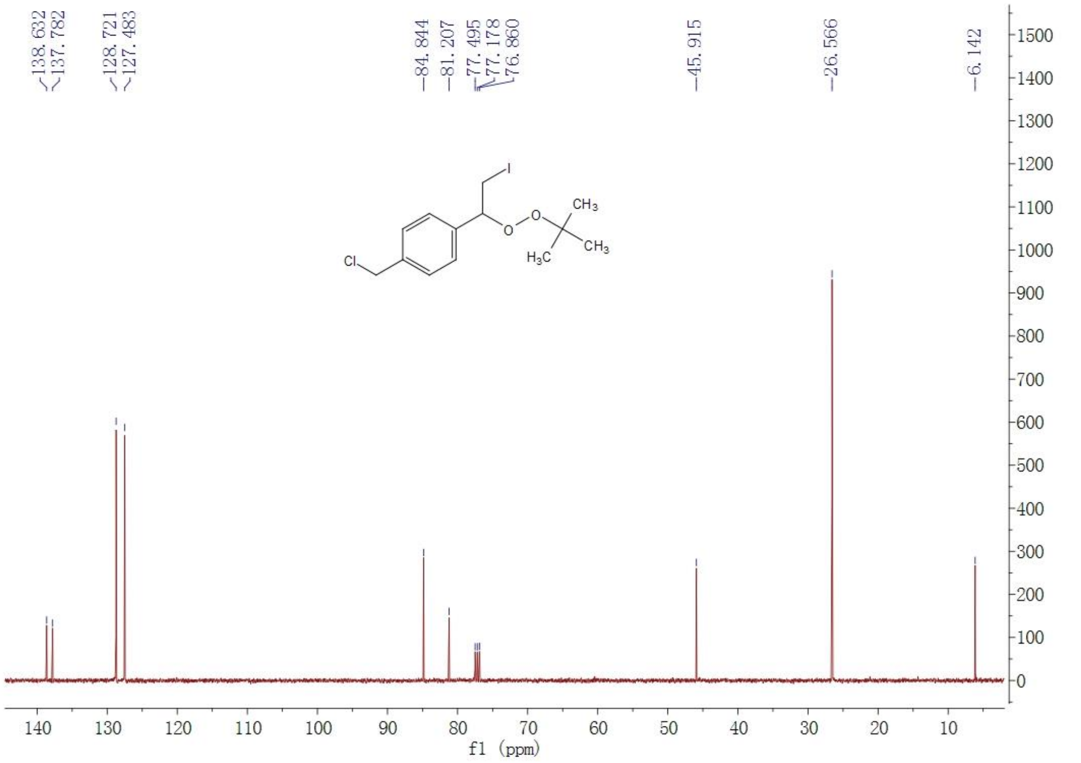

1-(*tert*-Butylperoxy)-2-iodo-1-methylcyclopentane (2t)

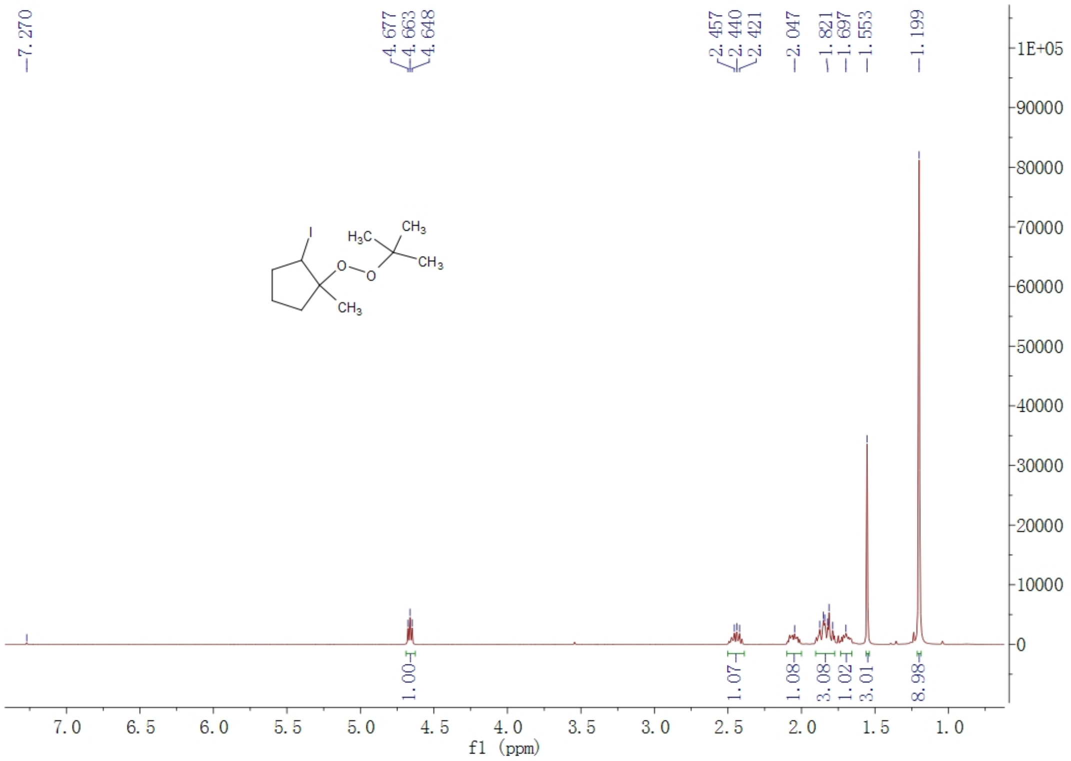

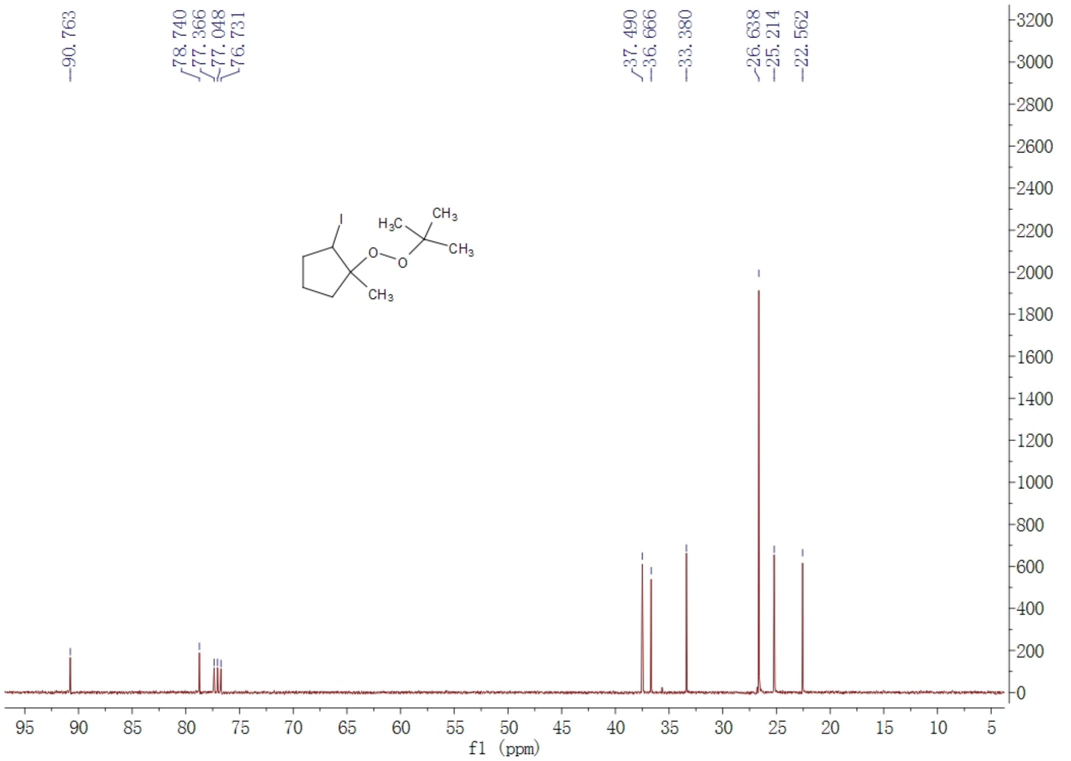

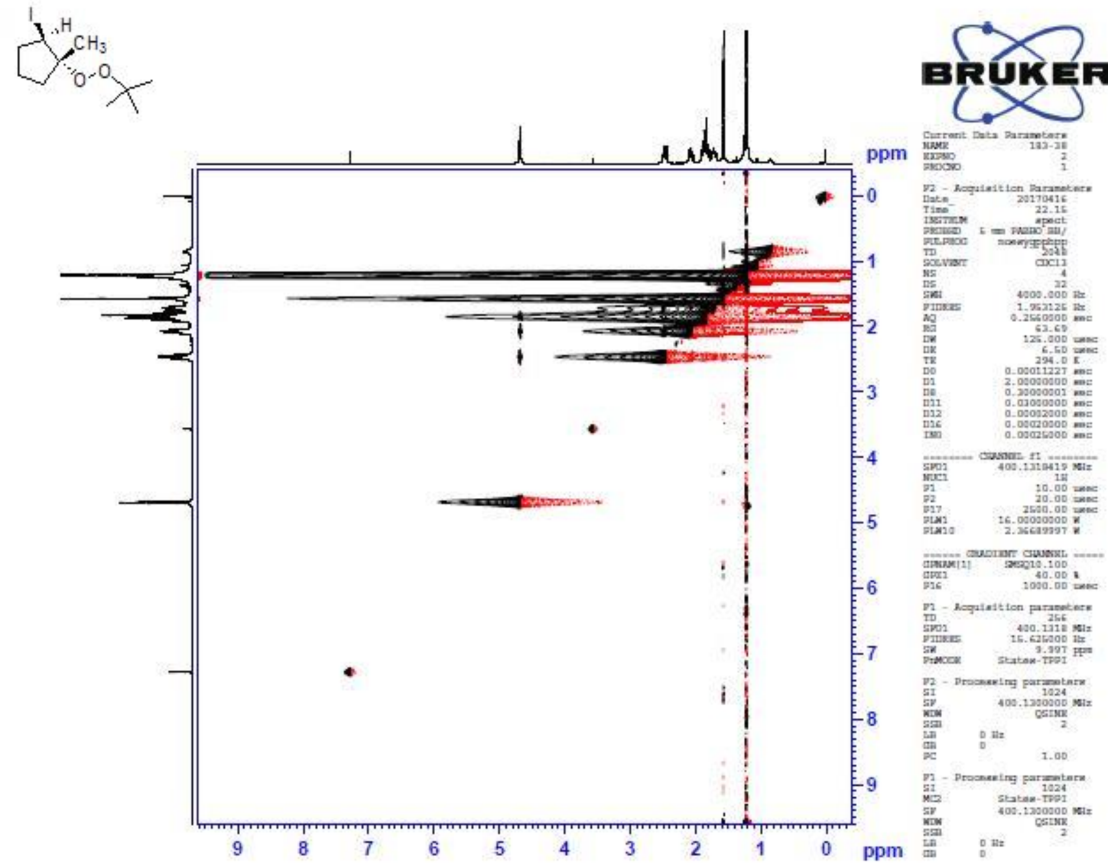

I
H
CH3
O
O
ppm
0
1
2
3
4
5
6
7
8
9
ppm
Current Data Parameters
NAME 183-38
EXPNO 2
PROCNO 1
F2 - Acquisition Parameters
Date_ 20170416
Time 22.16
INSTRUM spect
SOLVENT CDCl3
NS 4
DS 32
SWH 4000.000 Hz
AQ 0.2560000 sec
RG 63.69
DW 125.000 usec
DE 6.50 usec
TE 294.0 K
D1 2.00000000 sec
IN0 0.00025000 sec
CHANNEL f1
SFO1 400.1318419 MHz
NUC1 1H
P1 10.00 usec
P2 20.00 usec
P17 2500.00 usec
PLW1 16.00000000 W
GRADIENT CHANNEL
GPZ1 40.00 %
P16 1000.00 usec
F1 - Acquisition parameters
TD 256
SFO1 400.1318 MHz
SW 9.997 ppm
FnMODE States-TPPI
F2 - Processing parameters
SI 1024
SF 400.1300000 MHz
WDW QSINE
SSB 2
LB 0 Hz
GB 0
PC 1.00
F1 - Processing parameters
SI 1024
MC2 States-TPPI
SF 400.1300000 MHz
WDW QSINE
SSB 2
LB 0 Hz
GB 0

References

[1] Bloodworth, A. J.; Bowyer, K. J. *J. Org. Chem.* **1986**, *51*, 1790–1793.
